# Comparative Analysis of the Temporal Impacts of Corticosterone and Simulated Production Stressors on the Metabolome of Broiler Chickens

**DOI:** 10.3390/metabo13020144

**Published:** 2023-01-18

**Authors:** Catherine L. J. Brown, Sarah J. M. Zaytsoff, Andrew N. Iwaniuk, Gerlinde A. S. Metz, Tony Montina, G. Douglas Inglis

**Affiliations:** 1 Lethbridge Research and Development Centre, Agriculture and Agri-Food Canada, Lethbridge, AB T1J 4B1, Canada; 2 Southern Alberta Genome Sciences Centre, University of Lethbridge, Lethbridge, AB T1K 3M4, Canada; 3 Canadian Centre for Behavioural Neuroscience, University of Lethbridge, Lethbridge, AB T1K 3M4, Canada; 4 Department of Neuroscience, University of Lethbridge, Lethbridge, AB T1K 3M4, Canada; 5 Department of Chemistry and Biochemistry, University of Lethbridge, Lethbridge, AB T1K 3M4, Canada

**Keywords:** poultry, glucocorticoid hormone, ^1^H-NMR spectroscopy

## Abstract

The impact of physiological stress on the metabolome of breast muscle, liver, kidney, and hippocampus was investigated in Ross 308 broiler chicks. Simulated on-farm stressors were compared to a corticosterone model of physiological stress. The three different stressors investigated were: (i) corticosterone at a dose of 15 mg/kg of feed; (ii) heat treatment of 36 °C and 40% RH for 8 h per day; and (iii) isolation for 1 h per day. Liver, kidney, breast muscle, and hippocampus samples were taken after 2, 4, 6, and 8 days of stress treatment, and subjected to untargeted ^1^H-nuclear magnetic resonance (NMR) spectroscopy-based metabolomic analysis to provide insights on how stress can modulate metabolite profiles and biomarker discovery. Many of the metabolites that were significantly altered in tissues were amino acids, with glycine and alanine showing promise as candidate biomarkers of stress. Corticosterone was shown to significantly alter alanine, aspartate, and glutamate metabolism in the liver, breast, and hippocampus, while isolation altered the same pathways, but only in the kidneys and hippocampus. Isolation also significantly altered the glycine, serine, and threonine metabolism pathway in the liver and breast, while the same pathway was significantly altered by heat in the liver, kidneys, and hippocampus. The study’s findings support corticosterone as a model of stress. Moreover, a number of potential metabolite biomarkers were identified in chicken tissues, which may allow producers to effectively monitor stress and to objectively develop and evaluate on-farm mitigations, including practices that reduce stress and enhance bird health.

## 1. Introduction

Physiological stress has far-reaching and detrimental effects on all domestic animals, and the adverse impacts of stress on the health of birds is a major issue facing broiler chicken (*Gallus gallus domesticus*) production [1,2]. Chickens experience many different stressors, such as transport stress and fluctuations in temperature during the production cycle, which can elicit a stress response [3,4]. Stress reduces weight gain, increases the feed conversion ratio [5,6,7], reduces breast meat quality and carcass characteristics [8,9], and suppresses the immune system leaving birds susceptible to infections and ensuing disease [10,11,12]. It is not currently possible to monitor stress events before performance is affected in commercial flocks, and the identification of robust biomarkers of stress in chickens would benefit the chicken production industry as well as researchers investigating stress in chickens. A common biomarker of stress in poultry is corticosterone in blood [13]. However, corticosterone assays can be labor-intensive and costly [14]; are highly dependent on time of sampling [15,16], capture, handling, and restraint [15,17,18,19]; and lengthy sampling can lead to artificially high levels of corticosterone [20,21]. Furthermore, blood corticosterone concentration is an inaccurate measure of chronic stress [3,5,13,22].

An ideal biomarker should be sensitive enough to provide an early and robust indication of stress in birds, and allow for the implementation of mitigation strategies before there are any outward signs of distress, disease, or decreased performance [23]. Although there are many potential means of assessing stress, metabolomics has shown promise as a highly sensitive method of determining a stress condition based on relative concentrations of metabolites in biological samples [24]. In fact, recent nuclear magnetic resonance (NMR) spectroscopy-based metabolomics studies have shown that the metabolomes of eggs, breast muscle, liver, kidney, feces, and feathers can be used in a variety of ways to better understand domestic poultry physiology, including stress [6,25,26,27,28,29,30,31,32]. However, the use of metabolomics to identify biomarkers of health in poultry remains in its infancy.

In the current study, an untargeted NMR spectroscopy-based metabolomics approach was used to compare the corticosterone administration model of physiological stress to common simulated production stressors. We evaluated three different stressors which included: (i) corticosterone administered at a dose of 15 mg/kg of feed to simulate a physiological stress response [33,34,35]; (ii) heat treatment at 36 °C and 40% RH for 8 h per day [36,37,38,39,40]; and (iii) isolation for 1 h per day. Metabolic analyses of liver, kidney, breast muscle, and hippocampus samples at multiple time points were conducted. We hypothesize that the application of stress will result in metabolic changes in the tissues that are reflective of the systemic change in the birds due to their stress response. Furthermore, these changes will provide valuable insights into potential down-stream biomarkers that may be of use in the chicken production setting.

## 2. Materials and Methods

### 2.1. Experimental Design

The study was designed as a factorial experiment with four levels of stress treatment and four time points, arranged as a completely randomized design (Supplemental Figure S1). The four treatments were: (1) control (*n* = 24 chicks); (2) corticosterone stress (*n* = 24 chicks); (3) heat stress (*n* = 24 chicks); and (4) isolation stress (*n* = 24 chicks). The experiment was conducted on three separate occasions (i.e., runs), and each treatment time combination consisted of six replicate birds. Two replicate birds were included in each run.

### 2.2. Bird Husbandry

A local hatchery in Lethbridge provided the Ross 308 broiler chicks on the morning that they hatched (day 0). All chicks originated from a single broiler breeder farm, and originated from hens of known health status. Upon arrival at the Lethbridge Research and Development Centre (LeRDC) small animal facility, the chicks were randomly assigned to control, corticosterone, heat, or isolation treatments, and were acclimatized in pairs in individually ventilated cages (IVCs) (Techniplast, Montreal, QC, Canada). Chicks were provided ad libitum access to the same non-medicated starter diet (Hi-Pro Feeds, Lethbridge, AB, Canada) and sterile water [41]. During the brooding period, chicks were maintained at 30 °C for the first 2 days, 28 °C for the next 2 days, and at 26 °C for the remainder of the experimental period, with the exception of chicks subjected to heat stress. Birds were maintained on a 12 h light: 12 h dark cycle, and were weighed daily. Ambient conditions of temperature and humidity within animal rooms and within an IVC were continuously monitored using data loggers (GSP-6 Temperature and Humidity Data Logger, Elitech, San Jose, CA, USA).

### 2.3. Enteric Microbiota Establishment

To establish a uniform enteric microbiota (e.g., one that is representative of birds in a production facility), all chicks were orally administered cecal digesta containing enteric microorganisms on day 1, as described previously [32], with the exception that digesta harvested from the donor birds was frozen in a buffer containing glycerol (final concentration of 10% *w*/*v*). Briefly, digesta was thawed in an anoxic atmosphere, the slurry was transferred into sterile 3 mL syringes (1 mL per syringe), and the syringes were capped in order to prevent oxygen infiltration, and then maintained in a thermos that was pre-heated to 37 °C. Chicks were orally inoculated with the cecal digesta slurry within ≈20 min of removal of the syringes from the anoxic atmosphere. Each syringe was fitted with a flexible disposable PTFE feeding needle (15G × 3”, 2.8-mm-diameter ball) (Cadence Inc., Cranston, RI, USA). Individual chicks were gently restrained, their beaks were carefully opened by inserting the feeding needle, and the needle was carefully advanced into the mouth and then guided into the esophagus. Once in place, 1 mL of the cecal slurry was delivered into the esophagus, and the needle was carefully removed. Chicks were observed for 30 min, and none of the birds showed evidence of distress.

### 2.4. Stressors

Exposure of chicks to stressors commenced on day 5, and continued until the end of the experiment (Supplemental Figure S1). The birds assigned to the corticosterone treatment were provided feed containing 15 mg/kg corticosterone. The birds assigned to the heat treatment were placed in an adjacent room at 36 °C and 40% RH for 8 h per day to simulate a heat wave or ventilation breakdown in a broiler barn during a production cycle [36,37,38,39,40]. For the isolation treatment, the two birds co-housed in an individual IVC were separated into different animal rooms for 1 h per day. The two chicks had no line of sight and no physical contact with any other chicks in the room, and care was taken to ensure that ambient conditions within different rooms were equivalent [42]. Control treatment chicks were not exposed to experimental stressors.

### 2.5. Sample Collection

Randomly-selected chicks were sampled following 2, 4, 6, and 8 days of stress (i.e., day 6, 8, 10, and 12 of the experiment) (Supplemental Figure S1). At the defined endpoints, chicks were anesthetized with isoflurane (5% isoflurane; 1 L O_2_/min), and were then humanely euthanized by cervical dislocation under general anesthesia. Immediately after death, the head was removed, the cranium was opened, and the hippocampus was removed and snap-frozen in liquid nitrogen. The removal of the hippocampus tissue was based on the domestic chick brain atlas, and in all cases would have included the hippocampus proper as well as part of the adjacent parahippocampalis. Together they form the avian hippocampal formation [43,44]. Immediately after death, the abdomen was opened with a ventral midline incision, and a sample of the liver and one kidney were removed and snap-frozen. A skin incision over the breast muscle was then made, and a sample of muscle (pectoralis major) was removed and snap-frozen. All samples were stored at −80 °C until further processing.

### 2.6. Sample Preparation for NMR Spectroscopy

Amicon Ultra-0.5 centrifugal filters with a molecular weight cutoff of 3 kDa (Millipore Sigma, Oakville, ON, Canada) were used. Each filter was washed by adding 500 μL of Millipore water to the filter and centrifuging at 14,000× *g* for 5 min. This washing step was repeated ten times in order to ensure that all glycerol in the filter had been removed [45]. Approximately 150 mg of each sample was used. Metabolomics buffer (0.125 M KH_2_PO_4_, 0.5 M K_2_HPO_4_, 0.00375 M NaN_3_, and 0.375 M KF; pH 7.4) was added to the samples at a ratio of 2:1 (volume:mass) and the sample was homogenized using a Bullet Blender tissue homogenizer (Next Advance, Troy, NY, USA) with 150 mg of 2-mm-diameter zirconium oxide beads (Next Advance) on setting eight for 5 min. For breast tissue, which was more difficult to homogenize, the same procedure was followed, with the exception that homogenization was performed using a TissueLyser (Qiagen, Toronto, ON, Canada) and a single 6-mm-diameter steel bead for 10 min at 50 Hz. Samples were then centrifuged at 14,000× *g* for 5 min to pellet cellular debris, and 365 µL of the supernatant containing the small water-soluble metabolites was added to a pre-washed Amicon filter, along with 135 µL of metabolomics buffer. The filters were them centrifuged at 14,000× *g* for 30 min at 4 °C. For each sample, 360 µL of the filtrate was added to 140 μL of deuterium oxide containing 0.05% *v/v* trimethylsilylpropanoic acid (TSP) and 200 μL of metabolomics buffer (final total volume of 700 μL); TSP was used as a chemical shift reference for ^1^H-NMR spectroscopy. The solution was vortexed on the high setting, and then centrifuged at 12,000× *g* for 5 min at 4 °C to pellet any particulate matter. A 550 μL aliquot of the supernatant was placed in a 5 mm NMR tube, and the sample was run on a 700 MHz Bruker Avance III HD spectrometer (Bruker, Milton, ON, Canada) for spectral collection.

### 2.7. NMR Spectroscopy Data Acquisition and Processing

Metabolite spectra were obtained using the 1-D NOESY gradient pulse pre-saturation water suppression pulse sequence ‘noesygppr1d’ with 10 ms mixing time as previously described [30]. Briefly, each sample was run for 512 scans to a total acquisition size of 128 k, a spectral window of 20.5 ppm, a total data acquisition time of 4.56 s, a transmitter offset of ≈4.7 ppm, and a recycle delay of 1 s (total T1 relaxation recovery time of 5.56 s). The transmitter offset, utilized for water suppression was optimized prior to the start of data collection on each sample resulting in the reported offset of ≈4.7 ppm. The Bruker automation program “pulsecal” was used on each sample before data acquisition to guarantee that the 90-degree pulse was calibrated correctly, ensuring quantitative and comparable data across samples [46]. Prior to NMR spectroscopy data acquisition, three-dimensional and one-dimensional shimming experiments were conducted to correct for any variations in the static magnetic field. To ensure a minimal spectral resolution, a test spectrum was collected on each sample immediately following shimming. This test spectrum was subjected to line width measurements on the TSP peak at 50%, 25%, and 10% of the maximum height, and was required to meet a minimal line width of 1.0 Hz, 1.8 Hz, and 3.0 Hz, respectively. If this minimum specification was achieved, full data collection proceeded; if not, the shimming process was repeated until the specification was met. All measurements were recorded using a Bruker triple resonance TBO-Z probe at 22 °C. Spectra were initially processed using Bruker TopSpin software (v. 3.5 pl 7). The spectra were zero-filled to 256 k points, automatically phased using only zero-order phase correction, baseline-corrected using a first-order polynomial, and line-broadened using an exponential decay function of 0.3 Hz [47]. Spectra were then exported to MATLAB (MathWorks, Natick, MA, USA) as ASCII files, and underwent dynamic adaptive binning [48], followed by manual inspection and correction. Spectral binning resulted in 410, 496, 447, and 444 spectral bins for liver, kidney, breast muscle, and hippocampus samples, respectively. The dataset was normalized using the constant sum method, where each spectrum was set to have a unit total area, and each bin was a fraction of the total spectral integral (with the regions corresponding to water removed). The data were then log transformed and pareto-scaled to reduce the influence of intense peaks while emphasizing weaker ones. All peaks were referenced to TSP (0.00 δ) [46]. 

### 2.8. Statistical Analyses

The repeated measure within the mixed procedure of SAS (Statistical Analysis Software Institute Inc., Cary, NC, USA) was used to analyze daily weight gain in chicks; the appropriate covariance structure was selected according to the lowest Akaike’s Information Criterion (AIC). In conjunction with a significant F-test result, least square means were used to compare the stress and control treatments at individual sample times. 

Metabolite spectral bins were subjected to both univariate and multivariate analysis to determine which metabolites were significantly altered between stress and control treatments using MATLAB (MathWorks, Natick, MA, USA). The univariate measures were calculated using a decision tree algorithm as described by Goodpaster et al. [49]. The multivariate tests utilized the Variable Importance Analysis based on the random Variable Combination (VIAVC) algorithm, which combined both Partial Least Squares Discriminant Analysis (PLS-DA) and the area under the Receiver Operating Characteristics (ROC) curve to synergistically determine the best subset of metabolites for group classifications [50]. All *p*-values obtained for metabolomic analyses were Bonferroni–Holm corrected for multiple comparisons. MATLAB was also used to calculate the percent difference of the bins between treatments. The R package, MetaboanalystR (v. 2.0.1) [51] was used to carry out the Principle Component Analysis (PCA) and Orthogonal Partial Least Squares Discriminant Analysis (OPLS-DA). All OPLS-DA models were validated using double ten-fold cross-validation and permutation testing (2000 permutations) [52]. Metabolites were identified using Chenomx 8.2 NMR Suite (Chenomx Inc., Edmonton, AB, Canada), and the complete list of significant metabolites was used to carry out pathway topology using Metaboanalyst’s Metabolomics Pathway Analysis (MetPA) web-based tool (https://www.metaboanalyst.ca/) [53]. Pathway topology was conducted using the hypergeometric test for the over-representation analysis and relative betweenness centrality for the topology [54]; the KEGG database [55] for chicken pathways was utilized for this analysis. 

## 3. Results

### 3.1. Stressors Altered Weight Gain in Broiler Chickens

**Figure 1:**
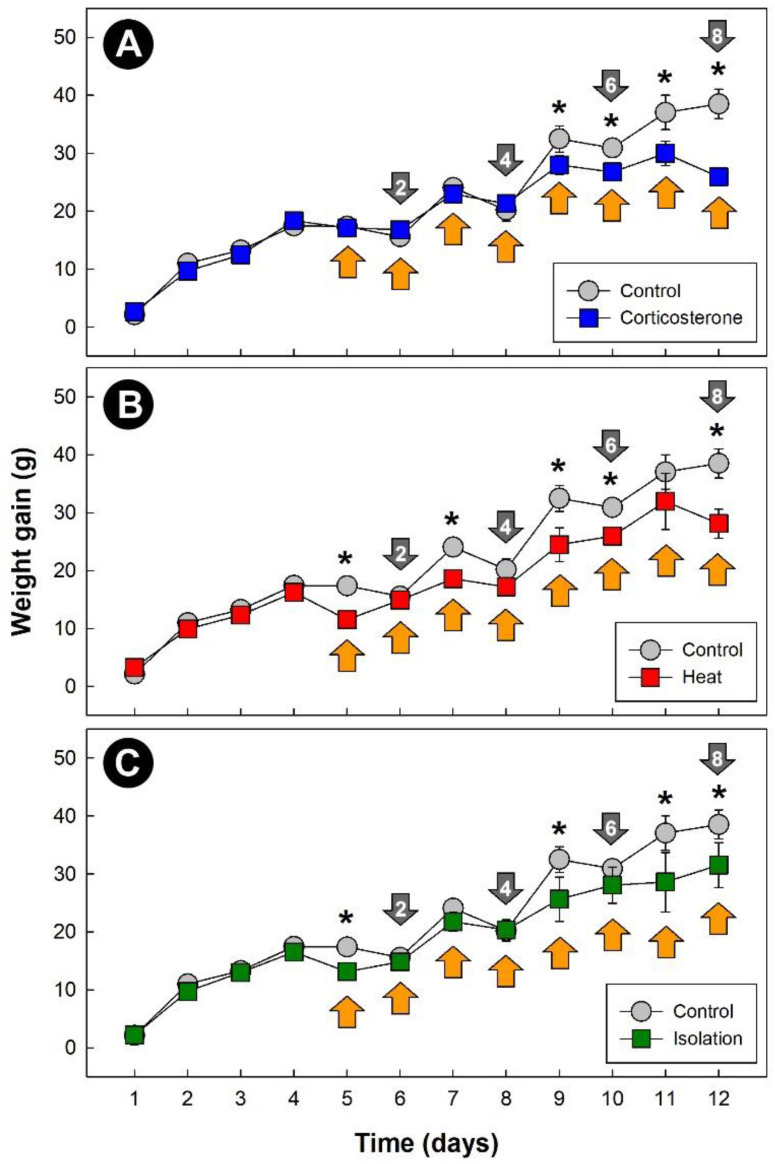
Daily weight gain of chicks (g). (**A**) Corticosterone treatment; (**B**) heat treatment (8 h at 36 °C per day); and (**C**) isolation treatment (1 h of isolation per day). Grey markers are control treatment birds. Markers indicated with an asterisk indicate a difference with the control treatment birds at individual time points (*p* ≤ 0.050). Orange arrows denote the stress exposure period, and grey arrows signify endpoints, with the number within the arrows denoting cumulative stress events. Vertical lines associated with markers are standard errors of the mean (SEM), and for instances where no vertical line is shown, the SEM was within the marker.

All three stressors temporally affected (*p* = 0.042) weight gain in chicks relative to control treatment birds (Figure 1A–C). Although the effects (*p* < 0.050) of stress on weight gain occurred primarily after 5 days of stress treatment, an immediate impact on weight gain due to both heat (Figure 1B) and isolation (Figure 1C) stress was observed.

### 3.2. Corticosterone Administration Substantively Altered the Liver, Kidney, Breast Muscle, and Hippocampus Metabolome

**Figure 2:**
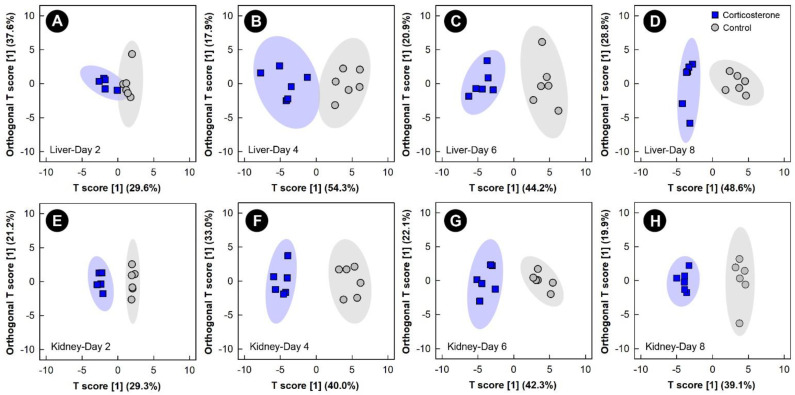
Orthogonal Partial Least Squares Discriminant Analysis (OPLS-DA) score plots of the liver and kidney metabolome of chicks administered corticosterone in their diet, or provided a diet free of glucocorticoid (control treatment), created using the spectral bins that were determined to be altered (*p* ≤ 0.050) by Variable Importance Analysis, based on random Variable Combination (VIAVC) analysis, and/or the univariate Mann–Whitney U test. (**A**) Liver at day 2; (**B**) liver at day 4; (**C**) liver at day 6; (**D**) liver at day 8; (**E**) kidney at day 2; (**F**) kidney at day 4; (**G**) kidney at day 6; (**H**) kidney at day 8. The *x*- and *y*-axis in the OPLS-DA plots represent the predictive (between-group separation) and orthogonal (within-group variation) components of the data, respectively. Each marker represents one bird (*n* = 6 per treatment), and shaded ellipsoids are the 95% confidence intervals for each treatment.

The multivariate VIAVC algorithm and/or univariate Mann–Whitney (MW) U-test revealed that metabolite bins were altered (*p* < 0.050) in the liver, kidney, breast muscle, and hippocampus in birds subjected to all three stressors relative to control treatment chicks. The significantly altered bins were then subjected to both unsupervised PCA and supervised OPLS-DA. Examination of all spectral bins for the corticosterone treatment showed no supervised or unsupervised group separation for any of the tissues (data not shown); therefore, an investigation into the multivariate modeling, using the bins identified as significantly altered by either the Mann–Whitney U-test and/or VIAVC, was carried out. This examination of the livers and kidneys in both corticosterone and control treatment birds showed supervised group separation, as well as good model fit (Q^2^) and variance explained by the model (R^2^), starting at 2 days and increasing at the 4, 6, and 8 endpoints (Figure 2, Supplemental Table S1). Relative quantities of specific metabolites differed (*p* ≤ 0.050) between the control and corticosterone treatments as determined by VIAVC analysis and/or MW U-test. For both the liver and kidney, there was also unsupervised separation between the corticosterone and control treatment birds at all endpoints (Supplemental Figure S2), with increasing separation observed with the duration of stress exposure.

**Figure 3:**
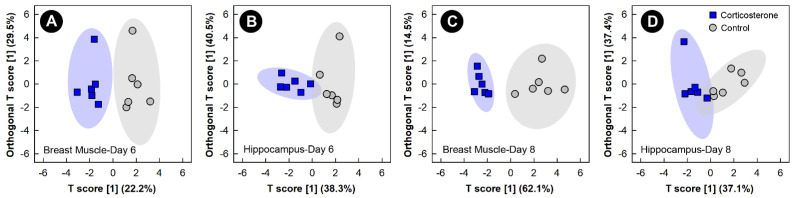
Orthogonal Partial Least Squares Discriminant Analysis (OPLS-DA) score plots of breast muscle and the hippocampus metabolome of chicks administered corticosterone in their diet, or provided a diet free of glucocorticoid (control treatment), created using the spectral bins that were determined to be altered (*p* ≤ 0.050) by Variable Importance Analysis, based on random Variable Combination (VIAVC) analysis, and/or the univariate Mann–Whitney U test. (**A**) Breast muscle at day 6; (**B**) hippocampus at day 6; (**C**) breast muscle at day 8; and (**D**) hippocampus at day 8. The *x*- and *y*-axis in the OPLS-DA plots represent the predictive (between-group separation) and orthogonal (within-group variation) components of the data, respectively. Each marker represents one bird (*n* = 6 per treatment), and shaded ellipsoids are the 95% confidence intervals for each treatment.

Supervised OPLS-DA analyses of breast muscle and hippocampus showed good model fit (Supplemental Table S1) and group separation of metabolite bins between control and corticosterone treatment birds, but only at the 6-day and 8-day endpoints (Figure 3). Supervised analyses for the 2-day and 4-day endpoints did not pass cross-validation or permutation testing, and the unsupervised PCA analyses of all endpoints did not show any clear group separation (data not shown).

### 3.3. Heat Stress Altered the Liver, Kidney, and Hippocampus Metabolome, but Not the Breast Muscle Metabolome

**Figure 4:**
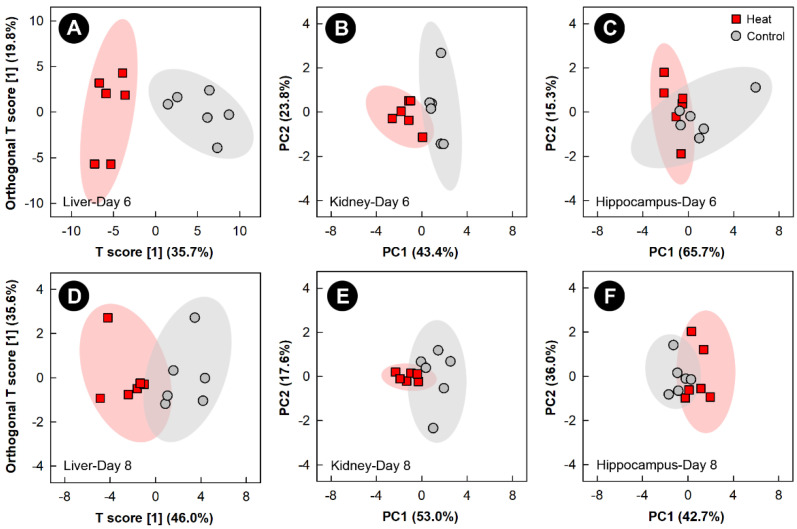
Orthogonal Partial Least Squares Discriminant Analysis (OPLS-DA) score plots of the liver metabolome of chicks exposed to heat stress relative to control chicks, created using the spectral bins which were determined to be altered (*p* ≤ 0.050) by Variable Importance Analysis, based on random Variable Combination (VIAVC) analysis, and/or the univariate Mann–Whitney U test. (**A**) Liver at day 6; (**D**) liver at day 8. The x- and *y*-axis in the OPLS-DA plots represent the predictive (between-group separation) and orthogonal (within group-variation) components of the data, respectively. Principal component analysis (PCA) score plots of the kidney and hippocampus metabolome of chicks exposed to heat stress relative to control chicks. (**B**) Kidney at day 6; (**C**) hippocampus at day 6; (**E**) kidney at day 8; and (**F**) hippocampus at day 8. The *x*- and *y*-axis of PCA plots show principal components one and two, respectively, with the number in brackets indicating the percent of variance explained by each component. Each marker represents one bird (*n* = 6 per treatment), and shaded ellipsoids are the 95% confidence intervals for each treatment.

Examination of all spectral bins for the heat treatment showed no supervised or unsupervised group separation for any of the tissues (data not shown). Examination of metabolite bins that were altered (*p* < 0.050) in the liver of heat treatment relative to control treatment birds, as determined by VIAVC analysis and/or the MW U test was completed using OPLS-DA analysis, and showed good model fit (Supplemental Table S1) and supervised group separation for the 6- and 8-day endpoints (Figure 4A,D). As supervised tests of the kidney and hippocampus did not pass permutation testing, unsupervised PCA analyses were used to compare the impact of heat exposure on these tissues relative to the control treatment at the 6- and 8-day endpoints (Figure 4B,C and Figure 4E,F, respectively). The PCA score plots of both tissues on both days showed good group separation, with the largest separation observed for the kidney at the 6-day endpoint. For breast muscle, neither supervised nor unsupervised separation was observed at any of the four endpoints due to heat treatment.

### 3.4. Isolation Stress Altered the Liver, Kidney, and Hippocampus Metabolome, but Not the Breast Muscle Metabolome

**Figure 5:**
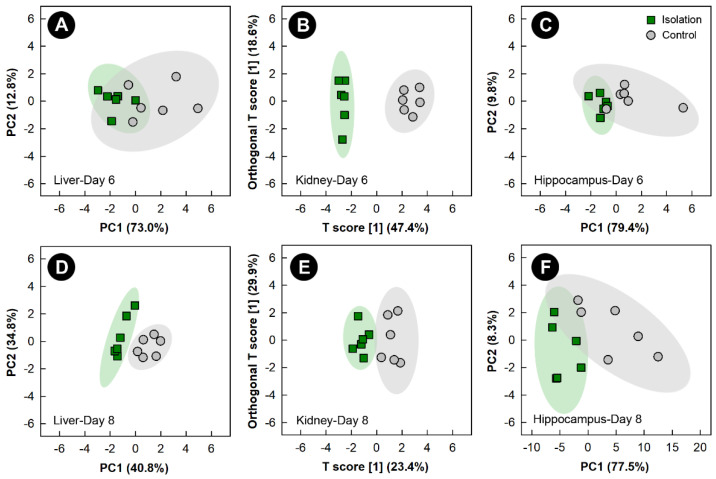
Principal component analysis (PCA) score plots of the liver and hippocampus metabolome of chicks exposed to isolation stress relative to control chicks. (**A**) Liver at day 6; (**C**) hippocampus at day 6; (**D**) liver at day 8; and (**F**) hippocampus at day 8. The x- and *y*-axis of PCA plots show principal components one and two, respectively, with the number in brackets indicating the percent of variance explained by each component. Orthogonal Partial Least Squares Discriminant Analysis (OPLS-DA) score plots of the kidney metabolome of chicks exposed to isolation stress, created using the spectral bins that were determined to be altered (*p* ≤ 0.050) by Variable Importance Analysis, based on random Variable Combination (VIAVC) analysis and/or the univariate Mann-Whitney U test. (**B**) Kidney at day 6; (**E**) kidney at day 8. The x- and *y*-axis of the OPLS-DA plots represent the predictive (between-group separation) and orthogonal (within-group variation) components of the data, respectively. Each marker represents one bird (*n* = 6 per treatment), and shaded ellipsoids are the 95% confidence intervals for each treatment.

Examination of all spectral bins for the isolation treatment showed no supervised or unsupervised group separation for any of the tissues (data not shown). PCA score plots of metabolite bins observed to be altered (*p* < 0.050) in the liver and hippocampus of isolation treatment relative to control treatment birds, as determined by VIAVC analysis and/or the Mann-Whitney U test showed unsupervised separation after 6 and 8 days of isolation (Figure 5A,C,D,F). Unsupervised separation of metabolite bins in the hippocampus and liver was also observed after 2 and 4 days of isolation treatment (Supplemental Figure S3). OPLS-DA analysis of metabolite bins from the kidney showed a good model fit (Supplemental Table S1) and supervised group separation between isolation and control treatment chicks at the 6- and 8-day endpoints (Figure 5B,E). In birds subjected to isolation stress, breast muscle did not show either supervised or unsupervised separation at any of the time points.

### 3.5. Different Metabolic Pathways Were Altered by Stressors

**Figure 6:**
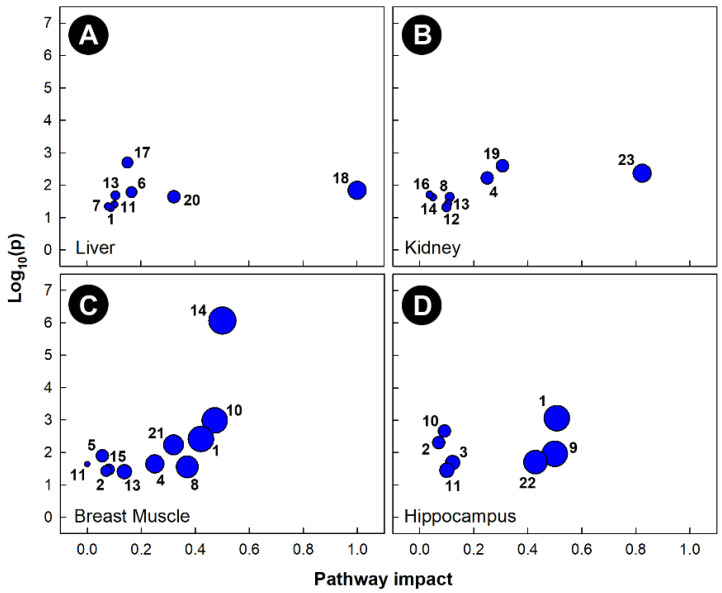
Pathway topologies of tissues from chicks subjected to corticosterone-incited stress. (**A**) Liver; (**B**) kidney; (**C**) breast muscle; and (**D**) hippocampus. Pathways are: 1, alanine, aspartate and glutamate metabolism; 2, arginine biosynthesis; 3, arginine and proline metabolism; 4, ascorbate and aldarate metabolism; 5, beta-alanine metabolism; 6, citrate cycle (TCA cycle); 7, galactose metabolism; 8, glutathione metabolism; 9, D-glutamine and D-glutamate metabolism; 10, glycine, serine and threonine metabolism; 11, glycolysis/gluconeogenesis; 12, glycerophospholipid metabolism; 13, glyoxylate and dicarboxylate metabolism; 14, histidine metabolism; 15, inositol phosphate metabolism; 16, nicotinate and nicotinamide metabolism; 17, pantothenate and CoA biosynthesis; 18, phenylalanine, tyrosine and tryptophan biosynthesis; 19, purine metabolism; 20, pyruvate metabolism; 21, starch and sucrose metabolism; 22, taurine and hypotaurine metabolism; and 23,vitamin B6 metabolism. Figures were created using Metabolanalyst’s Metabolomics Pathway Analysis (MetPA) web-based tool. The tool uses a hypergeometric test for over-representation analysis, and relative betweenness and centrality for topology; the KEGG database for chicken pathways was utilized for this analysis.

Pathway topology of chicks subjected to corticosterone-incited stress showed that multiple pathways were altered in all four of the tissues examined (Figure 6). The alanine, aspartate, and glutamate metabolism (KEGG gga00250), as well as the glycolysis and gluconeogenesis (KEGG gga00010) pathways were significantly altered in the liver, breast muscle, and hippocampus, but not in the kidney. The glyoxylate and dicarboxylate metabolism (KEGG gga00630) pathway was significantly altered in the liver, breast muscle, and kidney, but not in the hippocampus. Corticosterone administration significantly altered both the arginine biosynthesis (KEGG gga00220) and the glycine, serine, and threonine metabolism (KEGG gga00260) pathways in breast muscle and hippocampus, as well as both the ascorbate and aldarate metabolism (KEGG gga00053) and the glutathione metabolism (KEGG gga00480) pathways in kidney and breast muscle. The vitamin B6 metabolism (KEGG gga00750) pathway was altered only in the kidneys of birds administered corticosterone. The inositol phosphate metabolism (KEGG gga00562) and beta-alanine metabolism (KEGG gga00410) pathways were altered only in breast muscle, and the pyruvate metabolism (KEGG gga00620) and pantothenate and CoA biosynthesis (KEG gga00770) pathways were altered only in the livers of chicks administered corticosterone.

**Figure 7:**
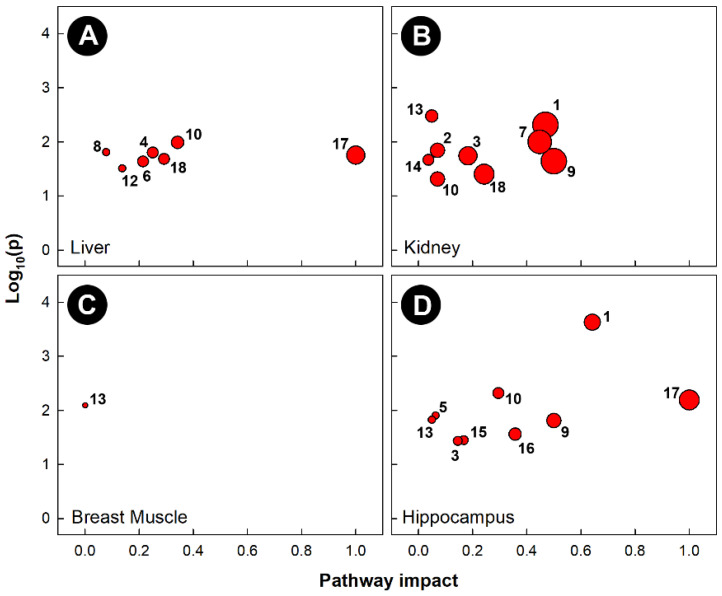
Pathway topologies of tissues from chicks subjected to heat stress. (**A**) Liver; (**B**) kidney; (**C**) breast muscle; and (**D**) hippocampus. Pathways are: 1, alanine, aspartate, and glutamate metabolism; 2, arginine biosynthesis; 3, arginine and proline metabolism; 4, ascorbate and aldarate metabolism; 5, butanoate metabolism; 6, citrate cycle (TCA cycle); 7, cysteine and methionine metabolism; 8, galactose metabolism; 9, D-glutamine and D-glutamate metabolism; 10, glycine, serine, and threonine metabolism; 11, glycolysis/gluconeogenesis; 12, glyoxylate and dicarboxylate metabolism; 13, histidine metabolism; 14, nicotinate and nicotinamide metabolism; 15, pentose phosphate pathway; 16, phenylalanine metabolism; 17, phenylalanine, tyrosine, and tryptophan biosynthesis; and 18, purine metabolism. See Figure 6 for a description of the analysis tools used.

Pathway topology of chicks subjected to heat stress also showed that different pathways were altered in the four tissues examined (Figure 7). Heat stress altered the glycine, serine, and threonine metabolism (KEGG gga00260) pathways in the liver, kidney, and hippocampus. The cysteine and methionine metabolism (KEGG gga00270) pathway was uniquely altered in kidneys, and the butanoate metabolism (KEGG gga00650) pathway was only altered in the hippocampus. Heat stress significantly altered the phenylalanine, tyrosine, and tryptophan biosynthesis (KEGG gga00400) pathways in the liver and hippocampus, the purine metabolism (KEGG gga00230) pathway in the liver and kidney, and both the arginine and proline metabolism (KEGG gga00330) and histidine metabolism (KEGG gga00340) pathways in the kidney and hippocampus. Heat treatment only altered a single pathway in the breast muscle (i.e., glycolysis and gluconeogenesis, KEGG gga00010).

**Figure 8:**
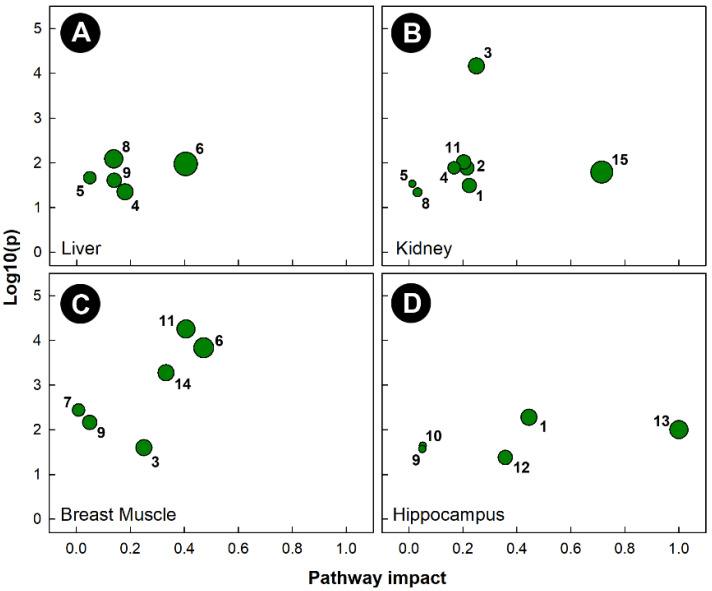
Pathway topologies of tissues from chicks subjected to isolation stress. (**A**) Liver; (**B**) kidney; (**C**) breast muscle; and (**D**) hippocampus. Pathways are: 1, alanine, aspartate, and glutamate metabolism; 2, amino sugar and nucleotide sugar metabolism; 3, ascorbate and aldarate metabolism; 4, citrate cycle (TCA cycle); 5, galactose metabolism; 6, glycine, serine, and threonine metabolism; 7, glycolysis/gluconeogenesis; 8, glyoxylate and dicarboxylate metabolism; 9, histidine metabolism; 10, nicotinate and nicotinamide metabolism; 11, pentose and glucuronate interconversions; 12, phenylalanine metabolism; 13, phenylalanine, tyrosine, and tryptophan biosynthesis; 14, starch and sucrose metabolism; and 15, taurine and hypotaurine metabolism. See Figure 6 for a description of the analysis tools used.

As with the other stressors, pathway topology showed that different pathways were altered in tissues harvested from chicks subjected to isolation stress (Figure 8). Isolation significantly altered the histidine metabolism (KEGG gga00340) pathway in the liver, breast muscle, and hippocampus, but not in the kidney. The amino sugar and nucleotide sugar metabolism (KEGG gga00520) pathway was uniquely altered in the kidneys of chicks subjected to isolation. Isolation also significantly altered the glycine, serine, and threonine metabolism (KEGG gga00260) pathways in the liver and breast muscle, the ascorbate and aldarate metabolism (KEGG gga00053) pathway in the kidney and breast muscle, the alanine, aspartate, and glutamate metabolism (KEGG gga00250) pathways in the kidney and hippocampus, and the glyoxylate and dicarboxylate metabolism (KEGG gga00630) pathways in the liver and kidney. As with heat stress, the only tissue in which the glycolysis and gluconeogenesis (KEGG gga00010) pathways were significantly altered was breast muscle.

### 3.6. Stressors Altered Relative Metabolite Concentrations in Tissues

The metabolite bins that were shown to be altered (*p* < 0.050) between the stress and control treatment chicks were subsequently identified using Chenomx, and the concentrations of a large number of metabolites were altered (*p* < 0.050) in liver, kidney, breast muscle, and hippocampus tissue due to the three stressors (Supplemental Table S2). In total, 106, 83, 69, and 90 unique metabolites were identified in the NMR spectra for kidney (Supplemental Table S3), liver (Supplemental Table S4), breast muscle (Supplemental Table S5), and hippocampus (Supplemental Table S6) tissues, respectively. 

**Figure 9:**
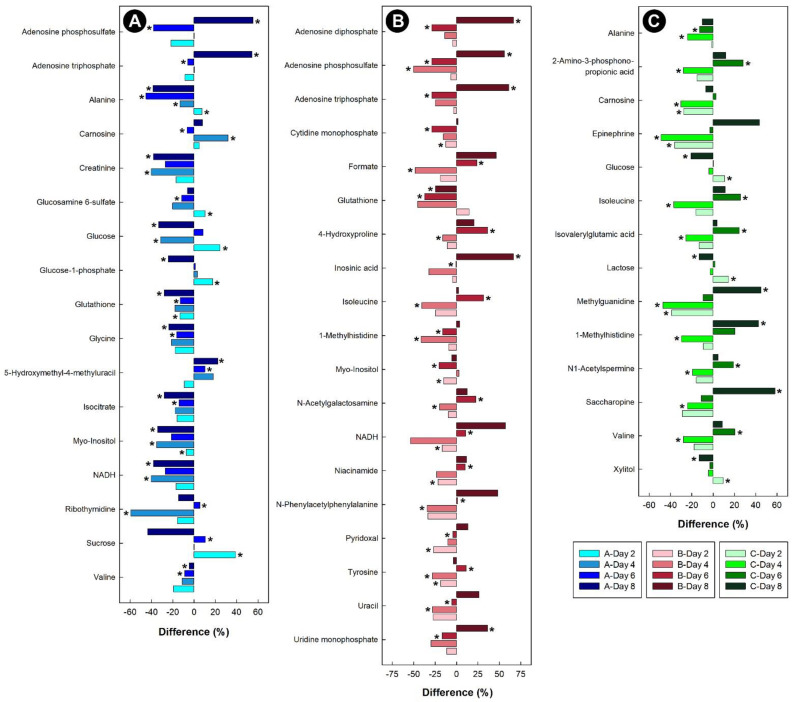
Regulation of common metabolites in the livers of broiler chicks subjected to stressors relative to control treatment at the four experimental endpoints. (**A**) Corticosterone (blue); (**B**) heat (red); and (**C**) isolation (green). Metabolites that were altered (*p* < 0.050) are indicated by asterisks.

Liver samples from birds administered corticosterone showed down-regulation of glycine and branched chain amino acids (BCAAs), such as valine (Figure 9A). While the amino acid alanine was initially up-regulated, the relative concentration dropped after 4 days of corticosterone and remained down-regulated in chicks at 6 and 8 days of corticosterone treatment. Glutathione, creatinine, NADH, myo-inositol, and isocitrate were all down-regulated in the livers of chicks treated with corticosterone. For the liver samples obtained from heat-stressed birds, myo-inositol was down-regulated, glutathione was initially up-regulated, and then down-regulated from day 4 onwards, and NADH, uracil, and 4-hydroxproline were initially down-regulated, and then up-regulated after heat treatment (Figure 9B). In the livers of chicks subjected to isolation stress, alanine was down-regulated, the BCAAs were initially down-regulated, and then increased after 6 days of isolation stress. The sugars lactose and glucose were initially up-regulated in chicks, and then down-regulated thereafter, and 1-methylhistidine was initially down-regulated, but increased in relative concentration after 6 and 8 days of isolation (Figure 9C).

**Figure 10:**
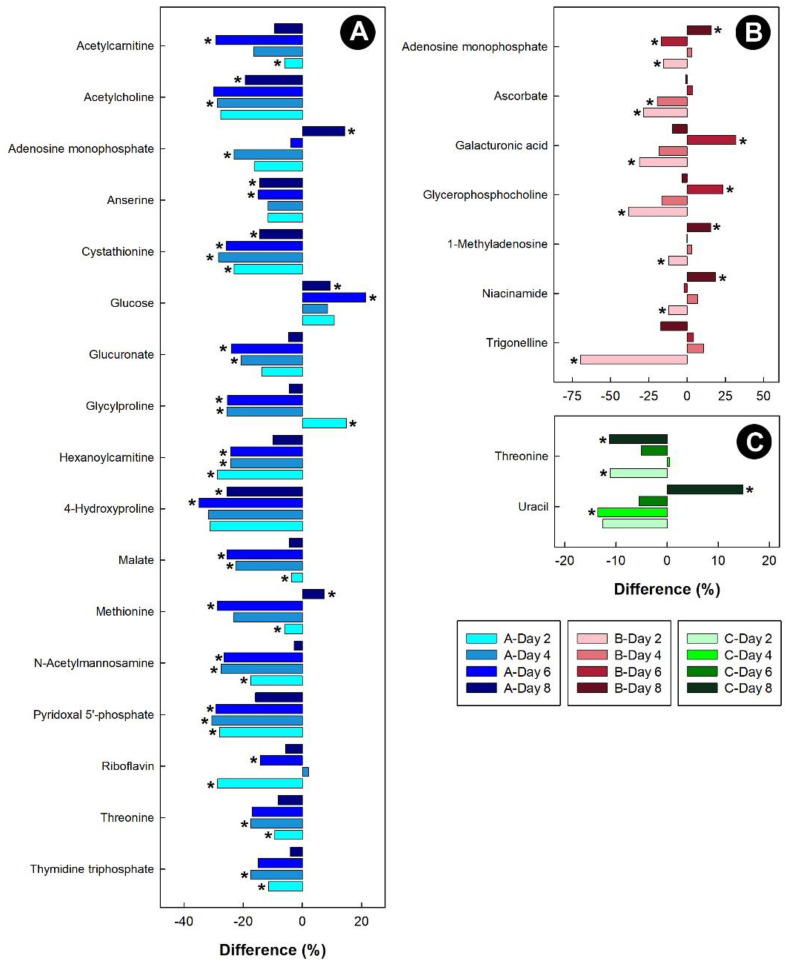
Regulation of common metabolites in the kidneys of broiler chicks subjected to stressors relative to the control treatment at the four experimental endpoints. (**A**) Corticosterone (blue); (**B**) heat (red); and (**C**) isolation (green). Metabolites that were altered (*p* < 0.050) are indicated by asterisks.

For the kidney samples obtained from birds administered corticosterone, threonine, malate, anserine, acetylcholoine, acetylcarnitine, and 4-hydroxyproline were all down-regulated (Figure 10A). Glucose was the only metabolite to be up-regulated in chicks at all time-points. For the heat treatment, 1-methyladenosine was initially down-regulated in chicks, and then up-regulated after 8 days of heat treatment (Figure 10B). Isolation resulted in the down-regulation of threonine in chicks, while uracil was initially down-regulated, and then up-regulated after 8 days in kidney tissue (Figure 10C).

**Figure 11:**
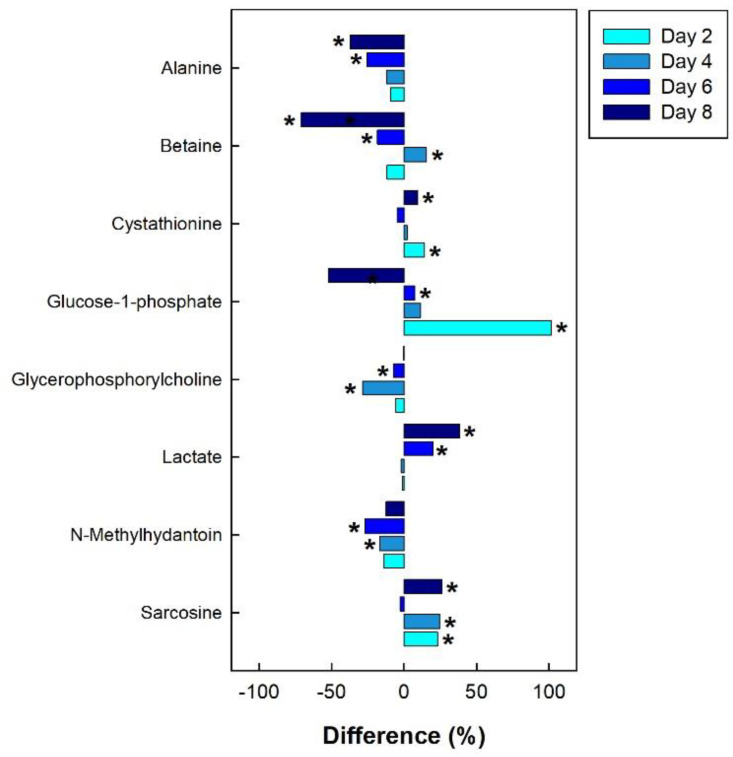
Regulation of common metabolites in the breast muscle of broiler chicks subjected to corticosterone-incited stress relative to the control treatment at the four experimental endpoints. Metabolites that were significantly altered (*p* < 0.050) are indicated by asterisks.

**Figure 12:**
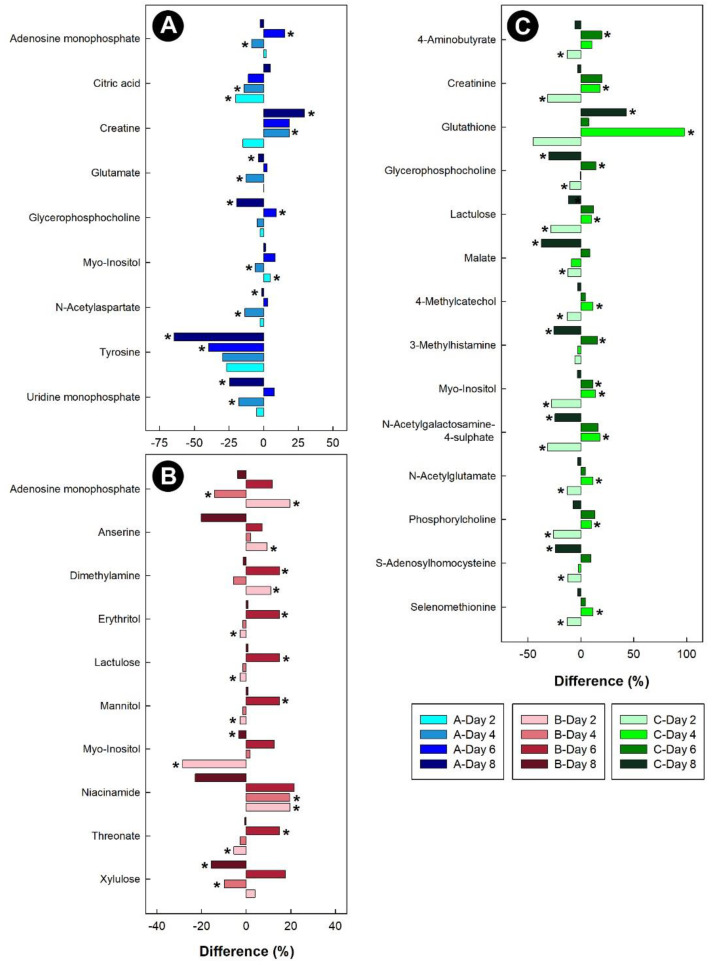
Regulation of common metabolites in the hippocampus of broiler chicks subjected to stressors relative to the control treatment at the four experimental endpoints. (**A**) Corticosterone (blue); (**B**) heat (red); and (**C**) isolation (green). Metabolites that were altered (*p* < 0.050) are indicated by asterisks.

In the breast muscle of chicks administered corticosterone, the amino acid alanine was down-regulated, along with glycero-phosphorylcholoine and N-methylhydantoin, while lactate and sarcosine were up-regulated (Figure 11). In the hippocampus of birds administered corticosterone, tyrosine and glutamate were down-regulated, and creatine was up-regulated (Figure 12A). For the heat treatment, anserine was initially up-regulated in the hippocampus of chicks after 2 days of heat treatment, and relative concentrations dropped over time thereafter (Figure 12B). In chicks subjected to isolation stress, glutathione was significantly up-regulated in the hippocampus after 4 and 8 days of isolation stress (Figure 12C).

## 4. Discussion

### 4.1. Overview

Metabolomics can provide comprehensive information on how animals respond to changes in environment, diet, pathophysiological stimuli, and genetic modification by providing a snapshot of all the metabolic activity of a biological system [56,57,58,59]. As a result of the adaptability of metabolomics, it is quickly becoming a favorite tool for the identification of biomarkers, particularly biomarkers of stress and disease [26,60,61]. In the current study, we observed distinct changes in the metabolomes of the liver, kidney, breast muscle, and hippocampus in response to three stressors. Moreover, all three stressors altered the concentrations of metabolites that have been linked to changes in both bird health and meat quality [32]. Although the three stressors examined altered different metabolites, the study’s findings showed that the corticosterone-incited physiological stress model mimicked the simulated production stressors (i.e., heat and isolation stress). Moreover, several of the metabolites that were differentially regulated due to stress (e.g., amino acids, including glycine, alanine, and anserine) may be promising biomarkers of stress in broiler chickens.

### 4.2. Glycine

The relative concentration of glycine in the liver decreased in birds that were administered corticosterone relative to control treatment chicks. The observed change in glycine concentrations corresponded to alterations in the glyoxylate and dicarboxylate metabolism pathway, which has previously been shown to be up-regulated in broilers subjected to heat stress [62]. Glycine is considered a Non-Essential Amino Acid (NEAA) because excess threonine and serine can interconvert to glycine [63], and generally, glycine is synthesized in sufficient quantities to meet demands from metabolic precursors [64]. However, as the immature livers of young birds often cannot produce adequate glycine to meet demand [63], glycine is considered to be essential in chicks for the first 8 weeks after hatch, with the highest demand occurring within the first 10 days of life [65,66]. Importantly, the broiler chicks used in the current study were ≤12 days old, and adequate glycine availability during this period is crucial.

Collagen is an important structural protein, the most abundant protein in the body, and approximately 30% of the amino acids comprising collagen are glycine residues [63]. The supplementation of diets with glycine during chick development can increase body weight, tarsus bone length, and beak width in birds later in life [63]. Thus, it is possible that the decrease in glycine that we observed in stressed birds will result in reduced availability of glycine for incorporation into collagen, which would correspond with the reduction in weight gain observed in chicks subjected to stressors in the current study. 

Glycine is a known oxidant scavenger, and it is essential for the regulation of the redox state of cells [31,67,68]. As well as acting as an oxidant scavenger on its own, glycine is also needed by chickens for the production of uric acid, which serves a dual purpose for nitrogen excretion [34,69,70,71] and as an antioxidant [72]. Birds dispose of nitrogenous waste from protein metabolism as uric acid (i.e., they are uricotelic) [73,74]. Avian species have an oxygen consumption rate that is approximately 2.5 times greater than mammals of similar size, and due to this fact, birds also have much higher plasma concentrations of uric acid to protect tissues from reactive-species damage; uric acid in broilers plasma is usually in the 0.2–0.8 mM range [71]. In birds, uric acid synthesis requires two nitrogen atoms from glutamine, a nitrogen atom from glutamate, and a nitrogen atom from glycine [75]. Corticosterone administration causes catabolism of protein tissues, such as skeletal muscles [76], and increases uric acid secretion [34], likely removing the nitrogenous waste resulting from protein catabolism. The decrease in glycine that we observed in chicks subjected to stress in the current study would thus be expected to result in a corresponding increase in uric acid production. 

In most animals including chickens, hydroxyproline is required for the synthesis of glycine. Moreover, up-regulation of hydroxyproline has been observed in response to many different kinds of stress, such as inflammatory and genotoxic stress [31,77,78]. In the current study, 4-hydroxyproline was initially down-regulated in the liver after 2 and 4 days of heat treatment, and then up-regulated after 6 and 8 days of heat stress. It is plausible that in the livers of birds subjected to heat stress, hydroxyproline was initially used to make up for the increased glycine demands. However after 4 days, demands for glycine were met, and hydroxyproline levels increased, possibly due to the acclimatization of birds to the elevated temperature. In the kidneys, but not the livers of chicks administered corticosterone, 4-hydroxyproline was down-regulated; however, glycine was correspondingly not down-regulated in the kidneys of birds subjected to corticosterone-incited stress. Thus, in stressed birds the kidney’s glycine requirements were met by heavily utilizing hydroxyproline. In contrast, the liver’s glycine demands were not met, and the liver did not use hydroxyproline to make up some of this deficiency. This generates questions about which enzymes can catalyze this reaction, and where they are located.

Reduced crude protein (CP) diets have become more popular for feeding chickens as a strategy to decrease environmental pollution emanating from chicken production, to increase litter quality, and to help reduce feed costs [79,80]. Glycine is sometimes an essential or a limiting NEAA, and increasing concentrations of glycine and other NEAAs in low CP chicken diets can be beneficial to stimulate feed intake and improve growth performance [64]. Conversely, too much dietary glycine can increase susceptibility to *Clostridium perfringens* infection [81]. Excess dietary glycine may also lead to decreased feed intake [81], as glycine administered directly to the chicken brain suppresses feed consumption [82]. 

Another important precursor for glycine is the amino acid serine. If serine is not converted to glycine, it can react with homocysteine to produce cystathionine, a reaction catalyzed by the enzyme cystathionine β-synthase (CBS). Deficiency in CBS activity can lead to increased concentrations of homocysteine and decreased bone quality, making chickens, and layers in particular, more susceptible to osteoporosis and bone breaks [83]. We observed that after 2 days of heat stress, homocysteine and cystathionine were up-regulated in breast muscle, which may have translated to increased CBS activity. Higher homocysteine levels in plasma are also linked to lower bone quality in layer chickens [84], and we observed that heat stress resulted in the down-regulation of both homocysteine and cystathionine in the kidneys of broiler chicks. The possible connection between stress and bone quality in young broiler chicks may warrant investigation, but metabolite biomarkers in blood as well as within tissues should be targeted. 

### 4.3. Alanine

Corticosterone administration resulted in the down-regulation of alanine in the liver and breast muscle. Moreover, alanine was down-regulated in the livers of chicks subjected to isolation stress. The alterations to alanine regulation led to significant alterations to the alanine, aspartate, and glutamate metabolism pathways. Stress causes a decrease in the concentrations of amino acids, particularly in the liver due to an increase in energy demands [2,31,85,86,87,88,89]. An increase in the alanine cycle would explain the drop in alanine observed in the breast muscle as it is exported from the muscles and transported to the liver to be converted into glucose [90]. If the alanine cycle were up-regulated in breast muscle, we would also expect to see alterations in pyruvate, lactate, and glucose, as well as changes in these three metabolites in the liver. In the current study, liver alanine was also down-regulated, which would follow an increase in the alanine cycle, where alanine is transported to the liver from muscles to be converted to glucose for export from the liver. However, the changes in liver glucose were unexpected; initially, glucose was up-regulated in birds administered corticosterone (i.e., at 2 days) and then drastically down-regulated. This may indicate that corticosterone caused an increased demand for glucose that was initially able to be met by an increase in the alanine cycle, but as the stress continued, the liver was not able to keep up with the increased glucose demands [87]. As glucose was not significantly altered in breast muscle, it is possible that glucose was taken up from the blood by other tissues. For example, we observed that glucose concentrations increased in the kidneys of broiler chicks after corticosterone administration. This result is in agreement with a previous study that also showed an increase in glucose in chicken kidneys under stress conditions [31].

Alanine catabolism is enhanced when there is an increased demand for TCA cycle intermediates (mainly pyruvate) for gluconeogenesis [64]. The administration of alanine prevents gluconeogenesis by inhibiting pyruvate kinase [66]. We only observed alterations in concentrations of alanine in liver and breast muscle, and in concentrations of lactate in breast muscle. This would suggest that an increase in the alanine cycle is not the sole contributor to the metabolite changes that we observed, and further research should be conducted to fully elucidate the impacts of stressors on alanine concentrations and the mechanisms involved. 

We observed that lactate was initially down-regulated in breast muscle after 2 and 4 days of corticosterone treatment, but was then up-regulated at the day 6 and day 8 endpoints. Lactate is an indicator of poor meat quality associated with a drop in pH and soft meat, which is often the result of rapid anaerobic glycolysis due to stress encountered during transport of broilers to abattoirs [91]. It is noteworthy that the change in lactate regulation that we observed corresponded with alterations to the glycolysis/gluconeogenesis pathway. 

### 4.4. Glutathione

Glutathione (GSH) is an important antioxidant that protects cells from potential oxidative damage and maintains homeostasis [66,92,93]. It is widely accepted that most of the commercially relevant stressors to which broilers are exposed in poultry barns (e.g., due to stocking density, handling, lighting, temperature, feed/water/nutrient imbalances, vaccination, drug administration, intestinal dysbiosis, and pathogen challenge) can cause oxidative stress [69]. Once reactive oxygen species (ROS)/reactive nitrogen species (RNS) concentrations become greater than the ability of the antioxidant defenses to neutralize them, oxidative stress occurs, and this can lead to biomolecules being damaged, which can result in impeded growth and development in poultry [69]. Selective breeding of broiler chickens for increased feed conversion and quick growth has concomitantly selected for increased sensitivity to oxidative stress [69]. GSH synthesis occurs in all cell types within the liver, which is the main producer and exporter of GSH [94]. GSH is formed from cysteine, glutamate, and glycine, with cysteine being the most limiting amino acid in the process [66,69,94]. We observed that cysteine was decreased in the hippocampus after 8 days in both corticosterone and heat stressed chicks, and GSH was up-regulated after 4 and 8 days of isolation stress. A possible alteration in the metabolism of GSH in the livers of chicks administered corticosterone in drinking water has previously been reported [31]. We observed that heat stress exposure initially up-regulated glutathione after 2 days, but glutathione was down-regulated after 4, 6, and 8 days of heat treatment. Heat stress impairs the mitochondrial thioreduction systems needed for GSH formation in growing chickens [69]. Reduced quantities of GSH in chicken livers suggests that the livers are either working more efficiently to protect the body from ROS/RNS, or that livers are having to work harder as a result of greater oxidant activity caused by stress [32]. 

### 4.5. Threonine

We observed that stress affected the regulation of threonine in broiler chicks. Threonine is an essential amino acid for broilers, and it is thought to be the third limiting dietary amino acid, behind lysine and sulfur-containing amino acids [95,96]. Threonine is a precursor for lysine and serine [96], and excess threonine and serine can be further converted to glycine [63]. Threonine is also an important component of feathers, comprising about 20% of the amino acids in feathers, along with serine [95]. Threonine is one of the few amino acids that must be supplied in the L-isomer form, as no D-amino acid oxidase or transaminase is present for interconverting the L- and D-isomers of threonine in animals [66]. Supplementing broiler feed with threonine has been shown to improve carcass characteristics and broiler performance [97]. It is also needed for the production of protective mucins in the digestive tract, and for proper immune function [96,97]. As corticosterone administration has an immunosuppressive effect on the innate immune system of chickens [98], the decrease in threonine that we observed in the kidneys of broiler chicks following corticosterone administration would be expected. We also observed a decrease in the concentration of threonine in the hippocampus of chicks subjected to isolation stress for 8 days. Down-regulation of threonine suggests that the amino acid was converted to glycine for the glycinergic neurotransmitter system. Glycine is an inhibitory transmitter involved in locomotion and some aspects of cognitive function [99]. How this might affect hippocampus function, which normally plays a role in spatial learning and memory [100] is unclear, but if it is a generalized effect across multiple brain regions, it could impact normal behavioral and neural development with uncertain consequences for adult behavior. 

### 4.6. Betaine

Betaine is a derivative of glycine that plays an important role in broiler metabolism by regulating growth, maintaining osmotic pressure, and acting as an antioxidant [91]. Betaine has also been shown to ameliorate bone quality in laying hens [84]. Dietary betaine supplementation can increase average daily gain in broilers, improve feed conversion ratios [91], and maintain meat quality under stress [91,101,102,103,104,105]. We observed that betaine was up-regulated in the breast muscle after 4 days of corticosterone treatment, but down-regulated thereafter. This suggests that chicks initially produced more betaine in response to stress; however over time, there was not enough betaine or betaine precursors such as glycine available to meet the demand of both the physiological and oxidative stress incited by corticosterone.

### 4.7. Breast Muscle Metabolome

Although heat and isolation stress affected the metabolome of other tissues, we observed that breast muscle was not appreciably affected by either heat or isolation stress. Early exposure to elevated temperatures helps birds adapt to heat later in life [106,107,108]. Initially, it was thought that brooding chicks did not experience stress from elevated temperatures, but it is now recognized that chicks are sensitive to heat stress [106,109,110,111]. The temperature that was selected in the current study (i.e., 36 °C) would be expected to elicit a thermal stress response in Ross 308 broilers during brooding as chicks as young as 7 days-of-age experience heat stress [112], and thermal stress after brooding occurs at 27 °C [113]. Stressors in this study were started at 5 days after hatching, and thus chicks should have experienced chronic heat stress particularly at the later endpoints. Whether prolonged exposure to heat and isolation stress would have imparted a greater impact on the metabolome of breast muscle as well as other muscles is uncertain.

High concentrations of circulating corticosterone suppress muscle growth and protein synthesis leading to decreased weight gain and increased muscle protein degradation [9,31,87,114,115], which likely contributed to the reduced weight gain that we observed in chicks administered corticosterone. In our previous research, we showed a much more pronounced effect of corticosterone on the breast muscle metabolome [6] than was observed in the current study. However, in our previous research, corticosterone was administered in drinking water at 10 and 30 mg/L; the 30 mg/L dose was twice that which was used in the current study [116,117,118]. Moreover, chickens drink more than they eat, which may have resulted in much higher exposures to corticosterone administered in water. Another difference with the study in which corticosterone was administered via drinking water was that the glucocorticoid was first dissolved in ethanol, and ethanol alone affected the breast muscle metabolome [31]. Thus, a possible interactive effect between ethanol and corticosterone may have contributed to the greater impact observed on the metabolome in our previous study. If the goal of research is to study the effects of physiological stress on the breast muscle metabolome of broiler chicks, a higher concentration of corticosterone should be used in in future studies.

### 4.8. Hippocampus Metabolome 

The hippocampus in birds is involved in spatial memory, and it is particularly sensitive to the adverse effects of stress [100]. We observed that isolation stress had the greatest impact on the metabolome of the hippocampus of broiler chicks. In contrast, the metabolome of the hippocampus was not particularly affected by either heat or corticosterone-incited stress, even though corticosterone is able to cross the chickens’ blood–brain barrier [119]. Under certain stress conditions, the hippocampus in birds is protected from incoming corticosterone, and although the mechanisms behind this are currently unknown [119], it is likely that protection is mediated by corticosteroid-binding globulins in the brain [120,121,122,123]. It is noteworthy that heat stress significantly altered the phenylalanine, tyrosine, and tryptophan biosynthesis pathways in the hippocampus, as tyrosine and tryoptophan are precursors for neurotransmitter production (i.e., dopamine and serotonin, respectively). Evidence obtained in the current study showed that targeting stress effects on the hippocampus metabolome has merit, but the full effects of stressors on the avian brain, including the identification of cardinal biomarkers will require the application of additional techniques beyond NMR spectroscopy, as well as sampling of a greater diversity of brain regions. 

## 5. Conclusions

Many metabolites were significantly affected by corticosterone and simulated on-farm stressors (heat and isolation stress) in broiler chicks, and effects varied across the tissues sampled. An ancillary goal of the research was to identify candidate metabolites for potential use as biomarkers of stress in broiler chickens that could be measured in more accessible downstream substrates, such as blood and feather pulp to allow producers to monitor their flocks for stress, to objectively evaluate mitigations, and to enhance production health. A number of amino acids, particularly glycine, 4-hydroxyproline, threonine, and alanine showed promise as cardinal metabolite biomarkers of stress. Threonine, which comprises 20% of the amino acids in feathers was down-regulated in the kidneys after 2 and 4 days of stress; this could be a promising acute biomarker in both blood and feather pulp. In addition, both 4-hydroxyproline and glycine were down-regulated, with the former occurring after 2 and 4 days, and the latter occurring after 6 and 8 days. These results suggest that 4-hydroxyproline may serve as a more sensitive biomarker of acute stress response, and glycine may be a more sensitive marker of chronic stress. Alanine was down-regulated in the liver after 4 and 6 days, and this suggests that alanine may be a potential biomarker of both subacute and chronic stress. Future research should validate these biomarkers using models of stress-precipitated disease, such as necrotic enteritis. Future studies should also validate potential biomarkers using a multifactorial approach that includes different nutritional regimes and genotypes.

## Data Availability

The data presented in this study are available upon request from the corresponding authors. Data is not publicly available due to privacy or ethical restrictions.

## Supplementary Materials

The following supporting information can be downloaded at: https://www.mdpi.com/article/10.3390/metabo13020144/s1, Figure S1: Treatments and the experimental timeline; Figure S2: Principle Component Analysis showing unsupervised multivariate analysis of the liver and kidney metabolome of chicks administered corticosterone in their diet or provided diet free of the glucocorticoid (control treatment); Figure S3: Principle Component Analysis showing unsupervised multivariate analysis of liver and hippocampus metabolome of chicks isolated for 1 h per day relative to control treatment chicks; Table S1: Model fit and *p*-values values for orthogonal partial least squares discriminant analysis score plots; Table S2: Number of significantly altered metabolites relative to the control treatment, as determined by the Mann–Whitney U test or the Variable Importance Analysis based on random Variable Combination algorithm for liver, kidney, breast muscle, and hippocampus; Table S3: Percent difference, *p*-values, and metabolites found to be significantly altered in chicken kidney after corticosterone, heat, and isolation treatments at 2, 4, 6, and 8 days as determined by the Mann–Whitney U test or Variable Importance Analysis based on random Variable Combination analysis; Table S4: Percent difference, *p*-values, and metabolites found to be significantly altered in chicken liver after corticosterone, heat, and isolation treatments at 2, 4, 6, and 8 days as determined by the Mann–Whitney U test or Variable Importance Analysis based on random Variable Combination analysis; Table S5: Percent difference, *p*-values, and metabolites found to be significantly altered in chicken breast muscle after corticosterone, heat, and isolation treatments at 2, 4, 6, and 8 days as determined by the Mann–Whitney U test or Variable Importance Analysis based on random Variable Combination analysis; Table S6: Percent difference, *p*-values, and metabolites found to be significantly altered in chicken hippocampus after corticosterone, heat, and isolation treatments at 2, 4, 6, and 8 days as determined by the Mann–Whitney U test or Variable Importance Analysis, based on random Variable Combination analysis.
