# Supplementary material for: Comparative Analysis of the Temporal Impacts of Corticosterone and Simulated Production Stressors on the Metabolome of Broiler Chickens
Source: Metabolites. 2023 Jan 18;13(2):144. doi: 10.3390/metabo13020144 (PMC9961940; doi:10.3390/metabo13020144)
Supplement: Supplementary file 1 [file metabolites-13-00144-s001.zip › metabolites-2015266-supplementary.pdf]

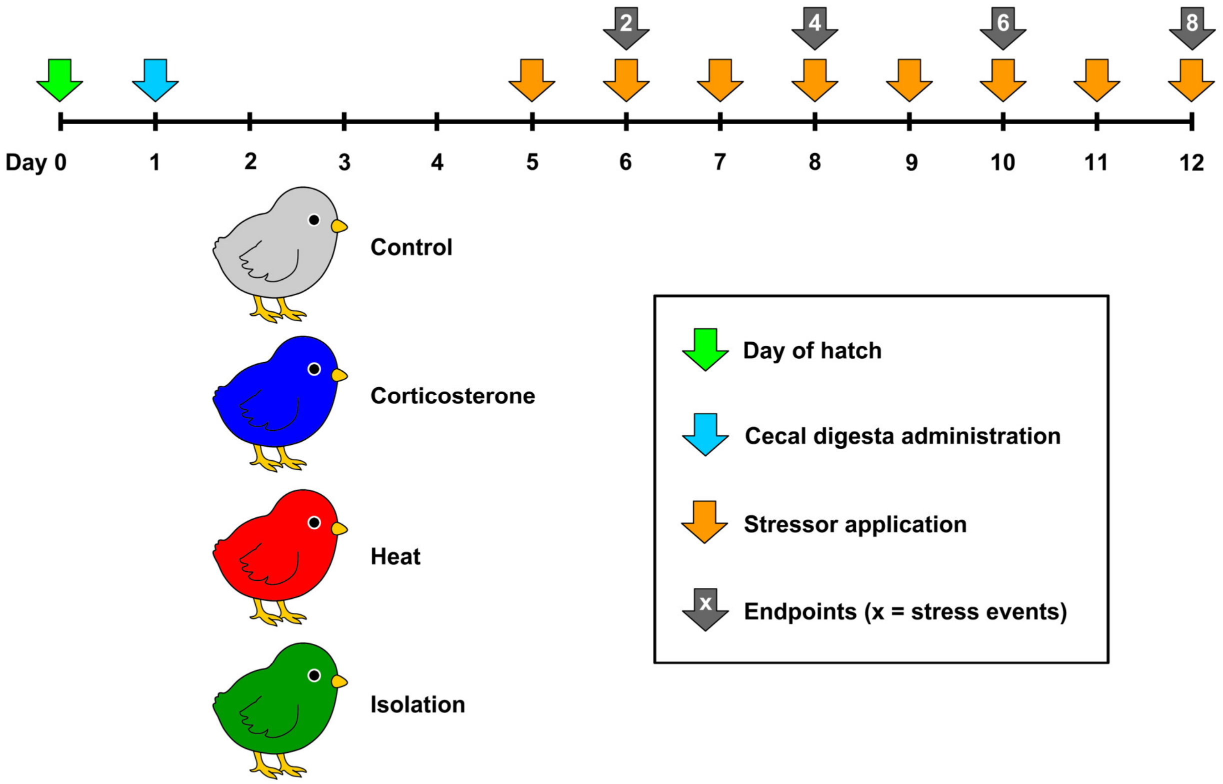

Figure S1. Treatments and the experimental timeline. The experiment was arranged as a factorial experiment with four levels of treatment and four levels of time. Each treatment-time point consisted of six replicate chicks (96 total).

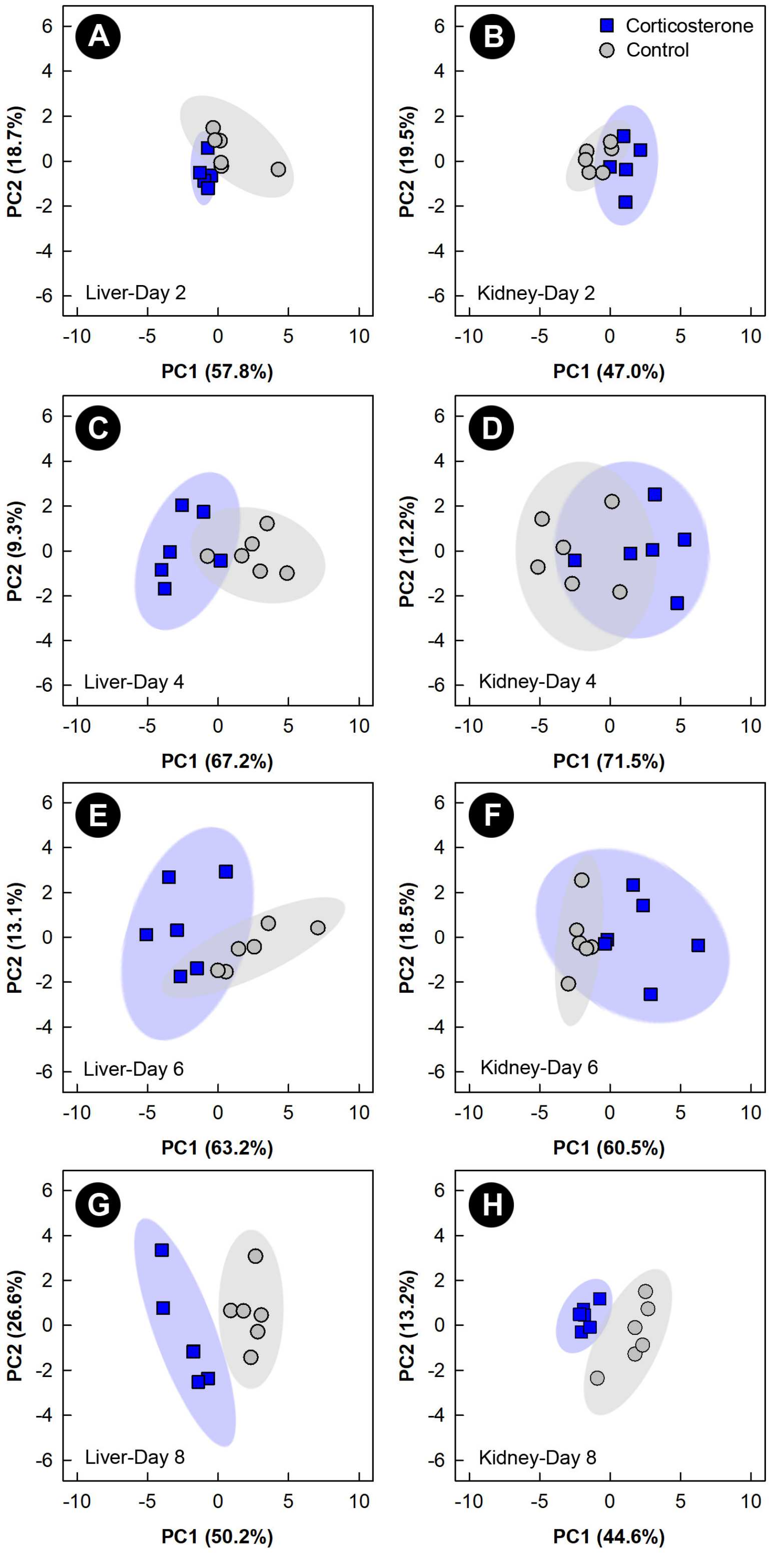

Figure S2. Principle Component Analysis (PCA) showing unsupervised multivariate analysis of the liver and kidney metabolome of chicks administered corticosterone in their diet or provided diet free of the glucocorticoid (control treatment). (**A**) Liver at; day 2; (**B**) kidney at day 2; (**C**) liver at day 4; (**D**) kidney at day 4; (**E**) liver at day 6; (**F**) kidney at day 6; (**G**) liver at day 8; and (**H**) kidney at day 8. The x-axis represents principle component 1, and the y-axis represents principle component 1. Each square or circle represents one bird (n=6 per treatment), and shaded ellipsoids are the 95% confidence intervals for each treatment.

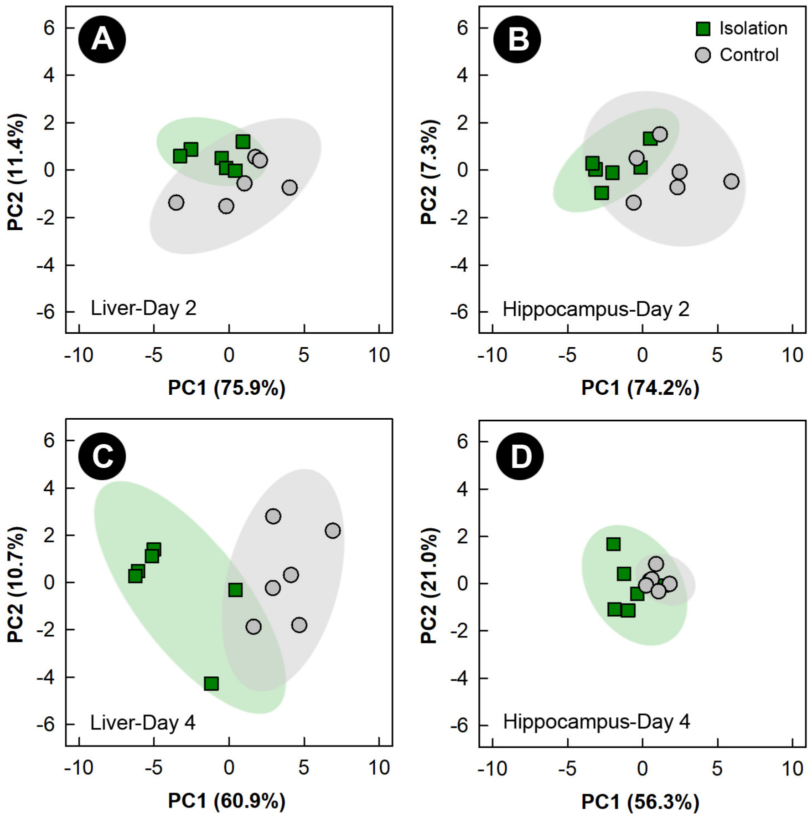

Figure S3. Principle Component Analysis (PCA) showing unsupervised multivariate analysis of liver and hippocampus metabolome of chicks isolated for 1 hr per day relative to control treatment chicks. (**A**) liver at day 2; (**B**) hippocampus at day 2; (**C**) liver at day 4; and (**D**) hippocampus at day 4. The x-axis represents principle component 1, and the y-axis represents principle component 1. Each square or circle represents one bird (n=6 per treatment), and shaded ellipsoids are the 95% confidence intervals for each treatment.

Table S1. Model fit and *p*-values values for Orthogonal Partial Least Squares Discriminant Analysis (OPLS-DA) score plots.

Figure	Tissue	Comparison	Q^2	*p*	R^2	*p*
2	Liver	CON-2[1] vs CORT-2[2]	0.473	0.039	0.844	0.007
		CON-2 vs CORT-4	0.675	0.016	0.864	0.048
		CON-6 vs CORT-6	0.836	0.004	0.939	0.007
		CON-8 vs CORT-8	0.912	0.004	0.948	0.004
	Kidney	CON-2 vs CORT-2	0.901	0.004	0.990	0.004
		CON-2 vs CORT-4	0.713	0.006	0.962	0.014
		CON-6 vs CORT-6	0.684	0.012	0.936	0.005
		CON-8 vs CORT-8	0.896	0.004	0.984	0.004
3	Breast muscle	CON-6 vs CORT-6	0.767	0.006	0.896	0.008
		CON-8 vs CORT-8	0.708	0.005	0.875	0.007
	Hippocampus	CON-6 vs CORT-6	0.739	0.002	0.826	0.018
		CON-8 vs CORT-8	0.538	0.018	0.736	0.013
4	Liver	CON-6 vs HEAT-6[3]	0.698	0.009	0.876	0.045
		CON-8 vs HEAT-8	0.479	0.022	0.757	0.022
5	Kidney	CON-6 vs ISOL-6[4]	0.865	0.002	0.975	0.002
		CON-8 vs ISOL-8	0.774	0.007	0.879	0.007

[1]Control treatment-day.
[2]Corticosterone treatment-day.
[3]Heat treatment-day.
[4]Isolation treatment-day.

Table S2. Number of significantly altered metabolites relative to the control treatment as determined by the Mann-Whitney U test (MW) or the Variable Importance Analysis based on random Variable Combination (VIAVC) algorithm for liver, kidney, and breast muscle, and hippocampus.

Tissue	Treatment	MW	VIAVC	MW + VIAVC
Liver	CORT-2	8	6	12
Liver	CORT-4	25	4	25
Liver	CORT-6	44	3	45
Liver	CORT-8	21	4	22
Liver	HEAT-2	6	5	11
Liver	HEAT-4	42	1	42
Liver	HEAT-6	50	15	55
Liver	HEAT-8	9	4	11
Liver	ISOL-2	7	11	17
Liver	ISOL-4	42	1	43
Liver	ISOL-6	8	3	11
Liver	ISOL-8	6	6	12
Kidney	CORT-2	9	11	17
Kidney	CORT-4	33	8	41
Kidney	CORT-6	14	4	17
Kidney	CORT-8	18	3	19
Kidney	HEAT-2	22	13	32
Kidney	HEAT-4	1	1	2
Kidney	HEAT-6	15	14	23
Kidney	HEAT-8	15	6	21
Kidney	ISOL-2	3	7	9
Kidney	ISOL-4	5	2	7
Kidney	ISOL-6	19	1	20
Kidney	ISOL-8	1	7	7
Breast muscle	CORT-2	4	9	12
Breast muscle	CORT-4	9	6	14
Breast muscle	CORT-6	6	14	16
Breast muscle	CORT-8	9	1	10
Breast muscle	HEAT-2	8	4	8
Breast muscle	HEAT-4	4	6	8
Breast muscle	HEAT-6	3	12	13
Breast muscle	HEAT-8	0	1	1
Breast muscle	ISOL-2	14	4	18
Breast muscle	ISOL-4	1	3	4
Breast muscle	ISOL-6	1	12	13
Breast muscle	ISOL-8	7	8	11
Hippocampus	CORT-2[1]	5	1	5
Hippocampus	CORT-4	28	2	28
Hippocampus	CORT-6	15	2	15
Hippocampus	CORT-8	9	3	9
Hippocampus	HEAT-2	11	18	29
Hippocampus	HEAT-4	1	6	7
Hippocampus	HEAT-6	8	8	16
Hippocampus	HEAT-8	7	3	9
Hippocampus	ISOL-2	26	4	27
Hippocampus	ISOL-4	7	5	14
Hippocampus	ISOL-6	5	3	8
Hippocampus	ISOL-8	39	3	39

[1]Corticosterone-day.
[2]Heat-day.
[3]Isolation-day.

Table S3. Percent difference, *p*-values, and metabolites found to be significantly altered in chicken kidney after corticosterone, heat, and isolation treatments at 2, 4, 6, and 8 days as determined by the Mann-Whitney U test (MW) or Variable Importance Analysis based on random Variable Combination analysis (VIAVC).

Treatment	Metabolite	Regulation	MW *p*-value	VIAVC *p*-value
CON-2[1] vs CORT-2[2]	Acetylcarnitine	-6.0	0.228	2.14E-06
CON-2 vs CORT-2	Cystathionine	-23.1	0.046	–
CON-2 vs CORT-2	Galactarate	-10.6	0.053	3.79E-09
CON-2 vs CORT-2	Glutamate	-4.0	0.374	2.81E-07
CON-2 vs CORT-2	Glycine	-5.8	0.427	2.87E-08
CON-2 vs CORT-2	Glycylproline	14.8	0.031	–
CON-2 vs CORT-2	Hexanoylcarnitine	-28.6	0.016	–
CON-2 vs CORT-2	Malate	-3.6	0.298	4.02E-08
CON-2 vs CORT-2	Methionine	-6.0	0.228	2.14E-06
CON-2 vs CORT-2	N-Acetylmannosamine	-17.5	0.126	6.45E-06
CON-2 vs CORT-2	N-Formyl-L-methionine	-26.4	0.036	–
CON-2 vs CORT-2	Phenylalanine	-11.7	0.131	2.06E-04
CON-2 vs CORT-2	Pyridoxal 5'-phosphate	-28.0	0.040	–
CON-2 vs CORT-2	Riboflavin	-28.6	0.016	–
CON-2 vs CORT-2	Threonine	-9.4	0.089	1.32E-05
CON-2 vs CORT-2	Thymidine triphosphate	-11.5	0.037	3.94E-05
CON-2 vs CORT-2	Trimethylamine N-oxide	19.6	0.032	–
CON-4 vs CORT-4	1-Methylhistidine	-23.9	0.028	–
CON-4 vs CORT-4	Acetylcholine	-28.6	0.019	–
CON-4 vs CORT-4	Adenosine monophosphate	-23.1	0.028	–
CON-4 vs CORT-4	Ascorbate	-27.9	0.026	–
CON-4 vs CORT-4	Citicoline	-27.5	0.007	–
CON-4 vs CORT-4	Citramalic acid	-23. 7	0.017	–
CON-4 vs CORT-4	Cystathionine	-28.3	0.015	–
CON-4 vs CORT-4	Deoxyadenosine triphosphate	-3.3	0.893	9.16E-06
CON-4 vs CORT-4	FADH	-24.3	0.045	–
CON-4 vs CORT-4	Galacturonic acid	-22.9	0.039	–
CON-4 vs CORT-4	Glucuronate	-20.7	0.017	–
CON-4 vs CORT-4	Glycerophosphocholine	-24.3	0.045	–
CON-4 vs CORT-4	Glycylproline	-25.6	0.023	–
CON-4 vs CORT-4	Guanosine monophosphate	-25.5	0.039	–
CON-4 vs CORT-4	Hexanoylcarnitine	-24.2	0.021	–
CON-4 vs CORT-4	Inosine triphosphate	-3.3	0.893	9.16E-06
CON-4 vs CORT-4	Inosinic acid	-20.7	0.018	–
CON-4 vs CORT-4	Isocitrate	-28.4	0.017	3.88E-05
CON-4 vs CORT-4	Isoleucine	3.1	0.770	6.50E-06
CON-4 vs CORT-4	Malate	-22.5	0.041	–
CON-4 vs CORT-4	N-Acetylcysteine	-29.7	0.012	–
CON-4 vs CORT-4	N-Acetylmannosamine	-27.5	0.007	–
CON-4 vs CORT-4	N-Acetylserine	-25.5	0.039	–
CON-4 vs CORT-4	NADP	-3.3	0.893	9.16E-06
CON-4 vs CORT-4	Niacinamide	-24.1	0.038	–
CON-4 vs CORT-4	O-Phosphocholine	-22.1	0.026	–
CON-4 vs CORT-4	Pyridoxal	-25.8	0.019	–
CON-4 vs CORT-4	Pyridoxal 5'-phosphate	-30.6	0.014	–
CON-4 vs CORT-4	Pyridoxamine	-22.2	0.030	–
CON-4 vs CORT-4	Riboflavin	2.0	0.779	5.94E-06
CON-4 vs CORT-4	Threonate	-18.5	0.015	3.16E-05
CON-4 vs CORT-4	Threonine	-17.4	0.017	–
CON-4 vs CORT-4	Thymidine triphosphate	-17.4	0.017	–

CON-4 vs CORT-4	Tryptophan	2.7	0.718	5.69E-08
CON-4 vs CORT-4	UDP-glucose	-20.7	0.018	–
CON-4 vs CORT-4	Unidentified	-42.4	0.025	–
CON-4 vs CORT-4	Unidentified	-24.3	0.013	–
CON-4 vs CORT-4	Unidentified	-26.1	0.012	–
CON-4 vs CORT-4	Uracil	-26.2	0.039	–
CON-4 vs CORT-4	Uridine monophosphate	-21.8	0.028	–
CON-6 vs CORT-6	3-Cresotinic acid	-22.9	0.023	–
CON-6 vs CORT-6	4-Hydroxyproline	-34.9	0.005	2.14E-09
CON-6 vs CORT-6	Acetylcarnitine	-29.3	0.041	–
CON-6 vs CORT-6	Anserine	-14.9	0.087	4.95E-06
CON-6 vs CORT-6	Cystathionine	-25. 8	0.016	3.28E-07
CON-6 vs CORT-6	Glucose	21.4	0.030	–
CON-6 vs CORT-6	Glucuronate	-24.0	0.057	–
CON-6 vs CORT-6	Glycylproline	-25.3	0.024	–
CON-6 vs CORT-6	Hexanoylcarnitine	-24.3	0.046	–
CON-6 vs CORT-6	Malate	-25.5	0.037	–
CON-6 vs CORT-6	Methionine	-28.7	0.008	3.28E-07
CON-6 vs CORT-6	N-Acetylmannosamine	-26.4	0.018	–
CON-6 vs CORT-6	Nicotinate	68.0	0.026	–
CON-6 vs CORT-6	N-Methylhydantoin	-29.0	0.030	–
CON-6 vs CORT-6	Pyridoxal 5'-phosphate	-29.3	0.041	–
CON-6 vs CORT-6	Unidentified	-34.3	0.025	–
CON-8 vs CORT-8	1,5-Anhydrosorbitol	-17.4	0.010	–
CON-8 vs CORT-8	4-Hydroxyproline	-25.6	0.006	–
CON-8 vs CORT-8	Acetylcholine	-19.3	0.032	–
CON-8 vs CORT-8	Adenosine diphosphate	25.1	0.023	–
CON-8 vs CORT-8	Adenosine monophosphate	14.3	0.039	–
CON-8 vs CORT-8	Adenosine triphosphate	14.3	0.039	–
CON-8 vs CORT-8	Anserine	-14.5	0.018	–
CON-8 vs CORT-8	Arginine	-15.3	0.020	–
CON-8 vs CORT-8	Cystathionine	-14.4	0.011	–
CON-8 vs CORT-8	Glucose	9.5	0.030	4.17E-05
CON-8 vs CORT-8	Hypotaurine	12.2	0.070	1.91E-05
CON-8 vs CORT-8	Methionine	7.4	0.027	–
CON-8 vs CORT-8	Phosphocholine	16.1	0.041	–
CON-8 vs CORT-8	Putrescine	9.6	0.045	–
CON-8 vs CORT-8	S-Adenosylhomocysteine	25.1	0.023	–
CON-8 vs CORT-8	Serine	9.4	0.030	4.17E-05
CON-8 vs CORT-8	Unidentified	30.8	0.026	–
CON-8 vs CORT-8	Valine	14.9	0.016	–
CON-8 vs CORT-8	Xanthine	40.4	0.041	–
CON-2 vs HEAT-2[3]	1-Methyladenosine	-12.0	0.011	–
CON-2 vs HEAT-2	2-Oxoglutarate	-17.7	0.304	8.46E-03
CON-2 vs HEAT-2	4-Pyridoxate	-16.6	0.329	1.97E-03
CON-2 vs HEAT-2	4-Pyridoxic acid	-50.8	0.041	–
CON-2 vs HEAT-2	5-Hydroxymethyl-4-methyluracil	-25.1	0.032	–
CON-2 vs HEAT-2	Adenosine monophosphate	-15.6	0.066	2.14E-02
CON-2 vs HEAT-2	Ascorbate	-28.7	0.028	–
CON-2 vs HEAT-2	Carnitine	-17.2	0.331	6.73E-03
CON-2 vs HEAT-2	Citicoline	-24.9	0.019	–
CON-2 vs HEAT-2	Cystine	16.0	0.104	6.77E-03
CON-2 vs HEAT-2	FAD	-24.9	0.235	2.48E-03
CON-2 vs HEAT-2	FADH	-37.7	0.015	–
CON-2 vs HEAT-2	Galactarate	-21.6	0.015	1.25E-03
CON-2 vs HEAT-2	Galacturonic acid	-31.0	0.026	–

CON-2 vs HEAT-2	Glucose	16.9	0.092	2.88E-03
CON-2 vs HEAT-2	Glucose-6-phosphate	17.2	0.099	3.68E-03
CON-2 vs HEAT-2	Glycerophosphocholine	-38.1	0.009	–
CON-2 vs HEAT-2	Inosinic acid	-15.2	0.108	1.48E-02
CON-2 vs HEAT-2	Lactulose	-31.8	0.012	–
CON-2 vs HEAT-2	Melatonin	-28.9	0.039	–
CON-2 vs HEAT-2	Methylimidazoleacetic acid	-103.9	0.014	–
CON-2 vs HEAT-2	N-Acetylmannosamine	-24.8	0.019	–
CON-2 vs HEAT-2	N-Formyl-L-methionine	-50.4	0.017	–
CON-2 vs HEAT-2	Niacinamide	-12.0	0.011	–
CON-2 vs HEAT-2	N-Methylnicotinamide	-20.3	0.047	–
CON-2 vs HEAT-2	Trans-Ferulic acid	-45.8	0.023	–
CON-2 vs HEAT-2	Trigonelline	-69.8	0.028	–
CON-2 vs HEAT-2	Tryptophan	-61.6	0.012	–
CON-2 vs HEAT-2	UDP-glucose	-15.6	0.066	2.14E-02
CON-2 vs HEAT-2	Unidentified	-22.1	0.162	2.44E-03
CON-2 vs HEAT-2	Uracil	-39.1	0.026	–
CON-2 vs HEAT-2	Uridine monophosphate	-27.1	0.032	1.07E-02
CON-4 vs HEAT-4	Ascorbate	-19.5	0.041	–
CON-4 vs HEAT-4	Lactate	-19.9	0.026	–
CON-6 vs HEAT-6	3-Methyladenine	-20.7	0.025	–
CON-6 vs HEAT-6	4-Hydroxyproline	-17.5	0.003	5.54E-08
CON-6 vs HEAT-6	Acetylcarnitine	-16.0	0.065	–
CON-6 vs HEAT-6	Adenosine monophosphate	-16.8	0.049	–
CON-6 vs HEAT-6	Aspartate	11.5	0.043	–
CON-6 vs HEAT-6	Betaine	7.6	0.052	–
CON-6 vs HEAT-6	Creatine	-9.0	0.048	1.36E-10
CON-6 vs HEAT-6	Creatinine	-9.0	0.048	1.36E-10
CON-6 vs HEAT-6	Cystathionine	-14.0	0.023	2.64E-05
CON-6 vs HEAT-6	Deoxyadenosine triphosphate	-20.7	0.025	–
CON-6 vs HEAT-6	Fructose	-17.3	0.284	1.28E-08
CON-6 vs HEAT-6	Galacturonic acid	31.8	0.093	1.14E-04
CON-6 vs HEAT-6	Glycerophosphocholine	23.5	0.015	2.07E-09
CON-6 vs HEAT-6	Guanidoacetate	-7.7	0.263	1.73E-11
CON-6 vs HEAT-6	Homocysteine	-11.5	0.047	–
CON-6 vs HEAT-6	L-Asparagine	-8.1	0.341	2.90E-07
CON-6 vs HEAT-6	Malic acid	-13.2	0.033	3.18E-05
CON-6 vs HEAT-6	Methionine	-14.0	0.023	2.64E-05
CON-6 vs HEAT-6	O-Phosphocholine	11.5	0.077	3.43E-05
CON-6 vs HEAT-6	Phosphocreatine	-9.0	0.048	1.36E-10
CON-6 vs HEAT-6	Pyridoxal 5'-phosphate	-16.0	0.065	–
CON-6 vs HEAT-6	Succinic acid	-44.5	0.012	2.29E-16
CON-6 vs HEAT-6	Xanthine	39.5	0.056	–
CON-8 vs HEAT-8	1-Methyladenosine	15.4	0.049	–
CON-8 vs HEAT-8	6-Phosphogluconic acid	-6.0	0.180	9.61E-11
CON-8 vs HEAT-8	Acetylglycine	11.3	0.079	8.38E-11
CON-8 vs HEAT-8	Adenosine diphosphate	16.2	0.041	–
CON-8 vs HEAT-8	Adenosine monophosphate	15.9	0.010	–
CON-8 vs HEAT-8	Adenosine triphosphate	15.9	0.010	–
CON-8 vs HEAT-8	Anserine	-13.6	0.045	–
CON-8 vs HEAT-8	Galactaric acid	-11.9	0.078	7.69E-13
CON-8 vs HEAT-8	Glutamate	9.8	0.079	8.11E-12
CON-8 vs HEAT-8	Glycerol	-15.4	0.047	–
CON-8 vs HEAT-8	Hypotaurine	15.9	0.028	6.67E-10
CON-8 vs HEAT-8	Methionine sulfoxide	19.3	0.048	–
CON-8 vs HEAT-8	Niacinamide	18.5	0.032	–

CON-8 vs HEAT-8	Nicotinate	20.1	0.019	–
CON-8 vs HEAT-8	Norspermidine	12.6	0.020	6.67E-10
CON-8 vs HEAT-8	S-Adenosylhomocysteine	16.2	0.041	–
CON-8 vs HEAT-8	Trigonelline	-17.1	0.030	–
CON-8 vs HEAT-8	Unidentified	21.0	0.040	–
CON-8 vs HEAT-8	Unidentified	-8.7	0.045	–
CON-2 vs ISOL-2[4]	1,5-Anhydrosorbitol	7.5	0.121	1.88E-25
CON-2 vs ISOL-2	7-Methyladenine	-21.7	0.045	5.95E-12
CON-2 vs ISOL-2	Adenosine monophosphate	8.4	0.340	3.56E-17
CON-2 vs ISOL-2	Adenosine triphosphate	8.4	0.340	3.56E-17
CON-2 vs ISOL-2	Citric Acid	11.2	0.058	7.90E-19
CON-2 vs ISOL-2	Formate	-22.2	0.026	–
CON-2 vs ISOL-2	Myo-Inositol	7.8	0.394	6.29E-16
CON-2 vs ISOL-2	Taurine	10.7	0.123	1.33E-61
CON-2 vs ISOL-2	Threonine	-11.1	0.046	–
CON-4 vs ISOL-4	Aspartate	-13.5	0.053	6.85E-128
CON-4 vs ISOL-4	Dimethylamine	-12.8	0.069	4.64E-128
CON-4 vs ISOL-4	Putrescine	-30.7	0.021	–
CON-4 vs ISOL-4	Serine	-17.2	0.036	–
CON-4 vs ISOL-4	Unidentified	30.4	0.032	–
CON-4 vs ISOL-4	Uracil	-13.5	0.033	–
CON-4 vs ISOL-4	Uridine monophosphate	20.8	0.046	–
CON-6 vs ISOL-6	3-Cresotinic acid	2.2	0.819	–
CON-6 vs ISOL-6	3-Methylhistamine	3.5	0.370	–
CON-6 vs ISOL-6	4-Hydroxy-3-methylbenzoic acid	25.8	0.589	–
CON-6 vs ISOL-6	4-Hydroxyproline	-4.5	0.457	–
CON-6 vs ISOL-6	Acetylcarnitine	-5.3	0.240	–
CON-6 vs ISOL-6	Cystathionine	-2.1	0.737	–
CON-6 vs ISOL-6	Glucarate	-2.6	0.759	–
CON-6 vs ISOL-6	Glucose	-1.3	0.844	–
CON-6 vs ISOL-6	Glucose-1-phosphate	3.0	0.518	–
CON-6 vs ISOL-6	Glucuronate	-4.7	0.533	–
CON-6 vs ISOL-6	Glycylproline	-5.6	0.514	–
CON-6 vs ISOL-6	Hexanoylcarnitine	-1.5	0.815	–
CON-6 vs ISOL-6	Malate	-4.3	0.619	–
CON-6 vs ISOL-6	Methionine	-2.1	0.737	–
CON-6 vs ISOL-6	N-Acetylmannosamine	-5.2	0.542	–
CON-6 vs ISOL-6	Nicotinate	9.8	0.582	–
CON-6 vs ISOL-6	N-Methylhydantoin	-4.9	0.583	–
CON-6 vs ISOL-6	Pyridoxal 5'-phosphate	-5.3	0.240	–
CON-6 vs ISOL-6	Riboflavin	-1.5	0.815	–
CON-6 vs ISOL-6	Succinic acid	-34.5	2.20E-04	1.14E-18
CON-8 vs ISOL-8	Anserine	-12.1	0.107	6.18E-13
CON-8 vs ISOL-8	Fructose	-10.5	0.342	4.57E-28
CON-8 vs ISOL-8	Galactarate	-12.4	0.211	3.82E-14
CON-8 vs ISOL-8	Hypotaurine	-8.4	0.318	7.11E-32
CON-8 vs ISOL-8	Threonine	-10.3	0.241	1.67E-13
CON-8 vs ISOL-8	Threonine	-12.4	0.211	3.82E-14
CON-8 vs ISOL-8	UDP-glucose	11.9	0.130	4.19E-12
CON-8 vs ISOL-8	Uracil	14.9	0.047	5.12E-20

[1]Control-day.
[2]Corticosterone-day.
[3]Heat-day.
[4]Isolation-day.

Table S4. Percent difference, *p*-values, and metabolites found to be significantly altered in chicken liver after corticosterone, heat, and isolation treatments at 2, 4, 6, and 8 days as determined by the Mann-Whitney U test (MW) or Variable Importance Analysis based on random Variable Combination analysis (VIAVC).

Treatment	Metabolite	Regulation	MW *p*-value	VIAVC *p*-value
CON-2[1] vs CORT-2[2]	Alanine	7.4	0.242	1.75E-09
CON-2 vs CORT-2	Arabinose	17.0	0.110	6.11E-05
CON-2 vs CORT-2	Fructose	12.2	0.176	3.05E-05
CON-2 vs CORT-2	Galacturonic acid	-27.9	0.052	–
CON-2 vs CORT-2	Glucosamine 6-sulfate	10.4	0.454	7.96E-06
CON-2 vs CORT-2	Glucose	24.5	0.079	6.11E-05
CON-2 vs CORT-2	Glucose-1-phosphate	17.5	0.248	6.29E-05
CON-2 vs CORT-2	Maltotriose	38.8	0.023	–
CON-2 vs CORT-2	Myo-Inositol	-7.4	0.355	4.99E-04
CON-2 vs CORT-2	Ribose	17.0	0.110	6.11E-05
CON-2 vs CORT-2	Sucrose	38.8	0.023	–
CON-4 vs CORT-4	5-Methylcytidine	-50.6	0.007	–
CON-4 vs CORT-4	Alanine	-13.4	0.000	6.15E-02
CON-4 vs CORT-4	Capryloylglycine	17.6	0.022	–
CON-4 vs CORT-4	Carnosine	31.9	0.018	–
CON-4 vs CORT-4	Creatinine	-40.3	0.001	3.41E-02
CON-4 vs CORT-4	FAD	17.7	0.046	–
CON-4 vs CORT-4	FADH	17.7	0.046	–
CON-4 vs CORT-4	Fumaric acid	39.6	0.006	–
CON-4 vs CORT-4	GABA	17.6	0.022	–
CON-4 vs CORT-4	Galactarate	39.3	0.003	–
CON-4 vs CORT-4	Glucose	-31.6	0.002	–
CON-4 vs CORT-4	Glutaconic acid	-46.4	0.035	–
CON-4 vs CORT-4	Glutathione	-17.9	0.040	–
CON-4 vs CORT-4	Homoserine	-17.9	0.040	–
CON-4 vs CORT-4	Hypotaurine	31.9	0.018	–
CON-4 vs CORT-4	Malate	34.5	0.008	–
CON-4 vs CORT-4	Myo-Inositol	-35.0	0.002	3.41E-02
CON-4 vs CORT-4	NADH	-40.3	0.001	3.41E-02
CON-4 vs CORT-4	Pyruvate	17.7	0.046	–
CON-4 vs CORT-4	Ribothymidine	-59.4	0.009	–
CON-4 vs CORT-4	Sebacic acid	-17.9	0.040	–
CON-4 vs CORT-4	Unidentified	-55.7	0.016	–
CON-4 vs CORT-4	Unidentified	19.5	0.019	–
CON-4 vs CORT-4	Ureidopropionic acid	23.7	0.026	–
CON-4 vs CORT-4	Valine	-28.8	0.002	–
CON-6 vs CORT-6	1-Methylhistidine	-4.7	0.751	–
CON-6 vs CORT-6	2-Amino-3-phosphonopropionic acid	-0.7	0.912	–
CON-6 vs CORT-6	4-Aminobutyrate	12.3	0.142	–
CON-6 vs CORT-6	5-Hydroxymethyl-4-methyluracil	10.1	0.225	–
CON-6 vs CORT-6	7-Methyladenine	-21.3	0.237	–
CON-6 vs CORT-6	Acetylcarnitine	-20.5	0.134	–
CON-6 vs CORT-6	Acetylcysteine	18.5	0.184	–
CON-6 vs CORT-6	Adenosine diphosphate	-18.8	0.441	–
CON-6 vs CORT-6	Adenosine phosphosulfate	-38.32	0.145	–
CON-6 vs CORT-6	Adenosine triphosphate	-5.8	0.644	–
CON-6 vs CORT-6	Alanine	-45.1	0.003	–
CON-6 vs CORT-6	Beta-N-Acetylglucosamine	13.1	0.567	–
CON-6 vs CORT-6	Carnitine	-2.0	0.757	–
CON-6 vs CORT-6	Carnosine	-6.7	0.675	–

CON-6 vs CORT-6	Cholate	3.9	0.852	–
CON-6 vs CORT-6	Choline	-19.5	0.233	–
CON-6 vs CORT-6	Coenzyme A	4.6	0.699	–
CON-6 vs CORT-6	Creatine	-2.9	0.966	–
CON-6 vs CORT-6	Epinephrine	-21.6	0.236	–
CON-6 vs CORT-6	Ethylmalonate	1.0	0.888	–
CON-6 vs CORT-6	Formate	-23.0	0.180	–
CON-6 vs CORT-6	Glucosamine 6-sulfate	-11.7	0.275	–
CON-6 vs CORT-6	Glucose	8.6	0.068	2.67E-04
CON-6 vs CORT-6	Glutathione	-13.0	0.279	–
CON-6 vs CORT-6	Glycerophosphocholine	9.1	0.023	2.14E-04
CON-6 vs CORT-6	Glycine	-16.4	0.030	–
CON-6 vs CORT-6	Glycylproline	-6.1	0.789	–
CON-6 vs CORT-6	Isocitrate	-14.1	0.241	–
CON-6 vs CORT-6	Isoleucine	-3.2	0.815	–
CON-6 vs CORT-6	Isovalerylglutamic acid	-6.6	0.519	–
CON-6 vs CORT-6	Lactic acid	-1.6	0.925	–
CON-6 vs CORT-6	Lysine	7.6	0.524	–
CON-6 vs CORT-6	Melatonin	3.1	0.763	–
CON-6 vs CORT-6	Methanol	13.4	0.229	–
CON-6 vs CORT-6	Methionine	-23.7	0.201	–
CON-6 vs CORT-6	Methylacetoacetic acid	-15.4	0.543	–
CON-6 vs CORT-6	Methylguanidine	-26.7	0.212	–
CON-6 vs CORT-6	N6-Acetyl-L-lysine	13.1	0.586	–
CON-6 vs CORT-6	N-Acetylglutamate	19.3	0.323	–
CON-6 vs CORT-6	N-Acetyllactosamine	1.3	0.926	–
CON-6 vs CORT-6	N-Acetylmannosamine	-3.8	0.310	–
CON-6 vs CORT-6	Nα-Acetyllysine	30.1	0.095	–
CON-6 vs CORT-6	Oxypurinol	1.3	0.730	–
CON-6 vs CORT-6	Pantothenic acid	9.6	1.000	–
CON-6 vs CORT-6	Phosphonoacetate	1.4	0.771	–
CON-6 vs CORT-6	Proline	19.2	0.247	–
CON-6 vs CORT-6	Riboflavin	-26.3	0.550	–
CON-6 vs CORT-6	Ribothymidine	5.5	0.467	–
CON-6 vs CORT-6	Sucrose	10.6	0.024	1.88E-04
CON-6 vs CORT-6	Tyrosine	-13.4	0.275	–
CON-6 vs CORT-6	Unidentified	2.6	0.968	–
CON-6 vs CORT-6	Unidentified	-23.2	0.390	–
CON-6 vs CORT-6	Unidentified	12.8	0.003	3.62E-04
CON-6 vs CORT-6	Unidentified	-6.3	0.700	–
CON-6 vs CORT-6	Valine	-9.1	0.692	–
CON-6 vs CORT-6	Xanthurenate	-62.4	0.068	–
CON-8 vs CORT-8	1,3-Dimethyluric acid	-27.9	0.004	–
CON-8 vs CORT-8	1-Methyladenine	56.7	0.061	–
CON-8 vs CORT-8	3-Phenyllactate	-46.5	0.014	–
CON-8 vs CORT-8	5-Hydroxymethyl-4-methyluracil	22.2	0.333	1.85E-02
CON-8 vs CORT-8	5-Methoxytryptamine	-46.5	0.014	–
CON-8 vs CORT-8	Acetoacetic acid	22.2	0.333	1.85E-02
CON-8 vs CORT-8	Adenosine phosphosulfate	55.2	0.038	–
CON-8 vs CORT-8	Adenosine triphosphate	54.2	0.012	–
CON-8 vs CORT-8	Creatinine	-38.2	0.001	–
CON-8 vs CORT-8	Glucuronic acid	-38.2	0.001	–
CON-8 vs CORT-8	Glucose	-33.3	0.009	–
CON-8 vs CORT-8	Glucose-1-phosphate	-24.3	0.041	–
CON-8 vs CORT-8	Glutathione	-27.9	0.025	–
CON-8 vs CORT-8	Glycine	-23.7	0.080	–

CON-8 vs CORT-8	Isocitrate	-27.9	0.025	–
CON-8 vs CORT-8	Alanine	-38.5	0.021	–
CON-8 vs CORT-8	Kynurenine	-41.3	0.032	–
CON-8 vs CORT-8	Phenylalanine	-43.92	0.023	–
CON-8 vs CORT-8	Valine	-4.6	0.169	1.85E-02
CON-8 vs CORT-8	Methionine sulfoxide	74.5	0.015	–
CON-8 vs CORT-8	Myo-Inositol	-34.1	0.005	1.89E-02
CON-8 vs CORT-8	NADH	-38.2	0.001	–
CON-2 vs HEAT-2[3]	Creatinine	-16.7	0.060	5.23E-24
CON-2 vs HEAT-2	Cytidine monophosphate	-12.6	0.104	6.16E-14
CON-2 vs HEAT-2	Erythrose	-23.1	0.032	–
CON-2 vs HEAT-2	Galactaric acid	-18.7	0.017	–
CON-2 vs HEAT-2	Glucarate	-21.2	0.023	–
CON-2 vs HEAT-2	Myo-Inositol	-15.4	0.088	1.44E-12
CON-2 vs HEAT-2	NADH	-16.7	0.060	5.23E-24
CON-2 vs HEAT-2	Niacinamide	-21.8	0.068	1.16E-09
CON-2 vs HEAT-2	Pyridoxal	-26.8	0.015	–
CON-2 vs HEAT-2	Tyrosine	-18.7	0.017	–
CON-2 vs HEAT-2	Unidentified	-23.2	0.017	–
CON-4 vs HEAT-4	1-Methylhistidine	-41.5	0.016	–
CON-4 vs HEAT-4	2-Amino-3-phosphonopropionic acid	-35.0	0.003	5.78E-04
CON-4 vs HEAT-4	3-Hexenedioic acid	-34.2	0.017	–
CON-4 vs HEAT-4	4-Hydroxyproline	-16.2	0.014	–
CON-4 vs HEAT-4	5-Hydroxymethyl-4-methyluracil	-17.4	0.023	–
CON-4 vs HEAT-4	7-Methyladenine	-36.0	0.025	–
CON-4 vs HEAT-4	Acetoacetate	-31.3	0.008	–
CON-4 vs HEAT-4	Acetylcarnitine	-27.4	0.017	–
CON-4 vs HEAT-4	Acetylcysteine	-43.7	0.022	–
CON-4 vs HEAT-4	Adenosine phosphosulfate	-50.3	0.025	–
CON-4 vs HEAT-4	Alanine	-17.7	0.019	–
CON-4 vs HEAT-4	Carnitine	-16.3	0.028	–
CON-4 vs HEAT-4	Choline	-36.1	0.006	–
CON-4 vs HEAT-4	Citrulline	-22.3	0.034	–
CON-4 vs HEAT-4	Creatine	-30.0	0.021	–
CON-4 vs HEAT-4	Formate	-48.4	0.004	–
CON-4 vs HEAT-4	Glutathione	-45.5	0.009	–
CON-4 vs HEAT-4	Glycine	-21.5	0.039	–
CON-4 vs HEAT-4	Glycylproline	-34.1	0.004	–
CON-4 vs HEAT-4	Isocitrate	-28.8	0.015	–
CON-4 vs HEAT-4	Isoleucine	-40.5	0.003	–
CON-4 vs HEAT-4	Isovalerylglutamic acid	-30.7	0.015	–
CON-4 vs HEAT-4	Lactic acid	-43.0	0.002	–
CON-4 vs HEAT-4	Methionine	-26.5	0.018	–
CON-4 vs HEAT-4	Methylacetoacetic acid	-18.9	0.026	–
CON-4 vs HEAT-4	N6-Acetyllysine	-40.9	0.001	–
CON-4 vs HEAT-4	N-Acetylgalactosamine	-19.9	0.029	–
CON-4 vs HEAT-4	N-Acetylglutamate	-31.5	0.003	–
CON-4 vs HEAT-4	N-Acetylmannosamine	-19.8	0.026	–
CON-4 vs HEAT-4	N-Phenylacetylphenylalanine	-34.5	0.038	–
CON-4 vs HEAT-4	Nα-Acetyllysine	-41.0	0.001	–
CON-4 vs HEAT-4	Oxypurinol	-52.0	0.003	–
CON-4 vs HEAT-4	Phosphocreatine	-27.3	0.016	–
CON-4 vs HEAT-4	Pyruvate	-16.7	0.025	–
CON-4 vs HEAT-4	Riboflavin	-48.6	0.009	–
CON-4 vs HEAT-4	Succinic acid	-33.7	0.031	–
CON-4 vs HEAT-4	Tyrosine	-28.3	0.007	–

CON-4 vs HEAT-4	Uracil	-28.1	0.017	–
CON-4 vs HEAT-4	Valine	-33.8	0.006	–
CON-4 vs HEAT-4	Xanthurenate	-32.4	0.015	–
CON-6 vs HEAT-6	1-Methylhistidine	-4.7	0.751	–
CON-6 vs HEAT-6	2-Amino-3-phosphonopropionic acid	-0.7	0.912	–
CON-6 vs HEAT-6	4-Aminobutyrate	12.3	0.142	–
CON-6 vs HEAT-6	5-Hydroxymethyl-4-methyluracil	10.1	0.225	–
CON-6 vs HEAT-6	7-Methyladenine	-21.3	0.237	–
CON-6 vs HEAT-6	Acetylcarnitine	-20.5	0.134	–
CON-6 vs HEAT-6	Acetylcysteine	18.5	0.184	–
CON-6 vs HEAT-6	Adenosine diphosphate	-18.8	0.441	–
CON-6 vs HEAT-6	Adenosine phosphosulfate	-38.3	0.145	–
CON-6 vs HEAT-6	Adenosine triphosphate	-5.8	0.644	–
CON-6 vs HEAT-6	Alanine	-45.1	0.003	–
CON-6 vs HEAT-6	Beta-N-Acetylglucosamine	13.1	0.567	–
CON-6 vs HEAT-6	Carnitine	-2.0	0.757	–
CON-6 vs HEAT-6	Carnosine	-6.7	0.675	–
CON-6 vs HEAT-6	Cholate	3.9	0.852	–
CON-6 vs HEAT-6	Choline	-19.5	0.233	–
CON-6 vs HEAT-6	Coenzyme A	4.6	0.699	–
CON-6 vs HEAT-6	Creatine	-2.9	0.966	–
CON-6 vs HEAT-6	Epinephrine	-21.6	0.236	–
CON-6 vs HEAT-6	Ethylmalonate	1.0	0.888	–
CON-6 vs HEAT-6	Formate	-23.0	0.180	–
CON-6 vs HEAT-6	Glucosamine 6-sulfate	-11.7	0.275	–
CON-6 vs HEAT-6	Glucose	8.6	0.068	2.67E-04
CON-6 vs HEAT-6	Glutathione	-13.0	0.279	–
CON-6 vs HEAT-6	Glycerophosphocholine	9.1	0.023	2.14E-04
CON-6 vs HEAT-6	Glycine	-16.4	0.030	–
CON-6 vs HEAT-6	Glycylproline	-6.1	0.789	–
CON-6 vs HEAT-6	Isocitrate	-14.1	0.241	–
CON-6 vs HEAT-6	Isoleucine	-3.2	0.815	–
CON-6 vs HEAT-6	Isovalerylglutamic acid	-6.6	0.519	–
CON-6 vs HEAT-6	Lactic acid	-1.6	0.925	–
CON-6 vs HEAT-6	Lysine	7.6	0.524	–
CON-6 vs HEAT-6	Melatonin	3.1	0.763	–
CON-6 vs HEAT-6	Methanol	13.4	0.229	–
CON-6 vs HEAT-6	Methionine	-23.7	0.201	–
CON-6 vs HEAT-6	Methylacetoacetic acid	-15.4	0.543	–
CON-6 vs HEAT-6	Methylguanidine	-26.7	0.212	–
CON-6 vs HEAT-6	N6-Acetyl-L-lysine	13.1	0.586	–
CON-6 vs HEAT-6	N-Acetylglutamate	19.3	0.323	–
CON-6 vs HEAT-6	N-Acetyllactosamine	1.3	0.926	–
CON-6 vs HEAT-6	N-Acetylmannosamine	-3.8	0.310	–
CON-6 vs HEAT-6	Nα-Acetyllysine	30.1	0.095	–
CON-6 vs HEAT-6	Oxypurinol	1.3	0.730	–
CON-6 vs HEAT-6	Pantothenic acid	9.6	1.000	–
CON-6 vs HEAT-6	Phosphonoacetate	1.4	0.771	–
CON-6 vs HEAT-6	Proline	19.2	0.247	–
CON-6 vs HEAT-6	Riboflavin	-26.3	0.550	–
CON-6 vs HEAT-6	Ribothymidine	5.5	0.467	–
CON-6 vs HEAT-6	Sucrose	10.6	0.024	1.88E-04
CON-6 vs HEAT-6	Tyrosine	-13.4	0.275	–
CON-6 vs HEAT-6	Unidentified	2.6	0.968	–
CON-6 vs HEAT-6	Unidentified	-23.2	0.390	–
CON-6 vs HEAT-6	Unidentified	12.8	0.003	3.62E-04

CON-6 vs HEAT-6	Unidentified	-6.3	0.700	–
CON-6 vs HEAT-6	Valine	-9.1	0.692	–
CON-6 vs HEAT-6	Xanthurenate	-62.4	0.068	–
CON-8 vs HEAT-8	1-Methyladenine	68.4	0.034	–
CON-8 vs HEAT-8	Adenosine diphosphate	66.4	0.008	–
CON-8 vs HEAT-8	Adenosine phosphosulfate	56.0	0.018	–
CON-8 vs HEAT-8	Adenosine triphosphate	61.0	0.021	–
CON-8 vs HEAT-8	Guanosine diphosphate	66.7	0.009	5.13E-05
CON-8 vs HEAT-8	Inosine	66.7	0.009	5.13E-05
CON-8 vs HEAT-8	Inosinic acid	66.4	0.008	–
CON-8 vs HEAT-8	N-Acetylgalactosamine	12.6	0.464	3.09E-05
CON-8 vs HEAT-8	Oxypurinol	38.9	0.019	–
CON-8 vs HEAT-8	Unidentified	-13.2	0.327	1.61E-05
CON-8 vs HEAT-8	Uridine monophosphate	36.2	0.047	–
CON-2 vs ISOL-2[4]	Betaine	14.3	0.050	–
CON-2 vs ISOL-2	Biocytin	-37.3	0.173	2.53E-05
CON-2 vs ISOL-2	Carnosine	-27.7	0.280	1.41E-07
CON-2 vs ISOL-2	Citric acid	-27.5	0.426	6.00E-10
CON-2 vs ISOL-2	Dimethylamine	-32.1	0.202	6.15E-24
CON-2 vs ISOL-2	Epinephrine	-36.2	0.201	4.69E-14
CON-2 vs ISOL-2	Glucose	10.8	0.270	4.23E-16
CON-2 vs ISOL-2	Glucose-1-phosphate	18.4	0.087	–
CON-2 vs ISOL-2	Glycyl-glycine	10.4	0.242	1.27E-15
CON-2 vs ISOL-2	Lactose	14.3	0.050	–
CON-2 vs ISOL-2	Methylguanidine	-38.9	0.134	4.32E-05
CON-2 vs ISOL-2	Myo-inositol	14.3	0.050	–
CON-2 vs ISOL-2	Sarcosine	-32.1	0.202	6.15E-24
CON-2 vs ISOL-2	Sucrose	7.2	0.375	5.26E-55
CON-2 vs ISOL-2	Taurine	18.9	0.057	1.03E-04
CON-2 vs ISOL-2	Trimethylamine N-oxide	23.5	0.082	–
CON-2 vs ISOL-2	Xylitol	9.3	0.209	5.41E-55
CON-4 vs ISOL-4	1-Methylhistidine	-29.6	0.030	–
CON-4 vs ISOL-4	2-Amino-3-phosphonopropionic acid	-28.1	0.004	–
CON-4 vs ISOL-4	4-Aminobutyrate	-17.0	0.016	–
CON-4 vs ISOL-4	5-Hydroxymethyl-4-methyluracil	-13.1	0.040	–
CON-4 vs ISOL-4	7-Methyladenine	-23.7	0.022	–
CON-4 vs ISOL-4	Acetylcarnitine	-26.2	0.017	–
CON-4 vs ISOL-4	Acetylcysteine	-51.5	0.011	–
CON-4 vs ISOL-4	Adenosine diphosphate	-44.4	0.021	–
CON-4 vs ISOL-4	Adenosine monophosphate	-49.1	0.046	–
CON-4 vs ISOL-4	Adenosine phosphosulfate	-40.7	0.041	–
CON-4 vs ISOL-4	Alanine	-24.0	0.008	–
CON-4 vs ISOL-4	Carnitine	-14.3	0.035	–
CON-4 vs ISOL-4	Carnosine	-30.3	0.012	–
CON-4 vs ISOL-4	Choline	-35.3	0.007	–
CON-4 vs ISOL-4	Creatine	-22.5	0.016	–
CON-4 vs ISOL-4	Epinephrine	-48.8	0.012	–
CON-4 vs ISOL-4	Formate	-39.4	0.010	–
CON-4 vs ISOL-4	Glucosamine 6-sulfate	-32.9	0.022	–
CON-4 vs ISOL-4	Glutathione	-45.4	0.021	–
CON-4 vs ISOL-4	Glycine	-18. 8	0.017	–
CON-4 vs ISOL-4	Glycylproline	-26.4	0.003	–
CON-4 vs ISOL-4	Isocitrate	-38.1	0.019	–
CON-4 vs ISOL-4	Isoleucine	-37.0	0.005	–
CON-4 vs ISOL-4	Isovalerylglutamic acid	-25.6	0.017	–
CON-4 vs ISOL-4	Lactic acid	-31.7	0.007	–

CON-4 vs ISOL-4	Melatonin	-23.4	0.003	–
CON-4 vs ISOL-4	Methanol	-23.8	0.015	–
CON-4 vs ISOL-4	Methionine	-24.1	0.016	–
CON-4 vs ISOL-4	Methylacetoacetic acid	-19.2	0.016	–
CON-4 vs ISOL-4	Methylguanidine	-47.3	0.026	–
CON-4 vs ISOL-4	N1-Acetylspermine	-19.5	0.021	–
CON-4 vs ISOL-4	N6-Acetyllysine	-34.6	0.000	–
CON-4 vs ISOL-4	N-Acetylglutamate	-22.7	0.006	–
CON-4 vs ISOL-4	N-Acetylmannosamine	-22.3	0.012	–
CON-4 vs ISOL-4	Nα-Acetyllysine	-35.0	0.000	–
CON-4 vs ISOL-4	Oxypurinol	-39.5	0.032	–
CON-4 vs ISOL-4	Phosphonoacetate	-25.1	0.049	–
CON-4 vs ISOL-4	Riboflavin	-54.5	0.017	–
CON-4 vs ISOL-4	Saccharopine	-24.0	0.066	2.66E-02
CON-4 vs ISOL-4	Tyrosine	-25.1	0.009	–
CON-4 vs ISOL-4	Valine	-27.9	0.008	–
CON-4 vs ISOL-4	Xanthurenate	-26.5	0.026	–
CON-6 vs ISOL-6	2-Amino-3-phosphonopropionic acid	28.3	0.013	–
CON-6 vs ISOL-6	Alanine	-12.9	0.003	5.43E-18
CON-6 vs ISOL-6	Dihydrothymine	83.3	0.001	1.48E-19
CON-6 vs ISOL-6	Glycerol	-12.9	0.003	5.43E-18
CON-6 vs ISOL-6	Isoleucine	25.8	0.023	–
CON-6 vs ISOL-6	Isovalerylglutamic acid	24.5	0.020	–
CON-6 vs ISOL-6	lysine	29.3	0.006	–
CON-6 vs ISOL-6	N1-Acetylspermine	19.1	0.011	–
CON-6 vs ISOL-6	N-Acetylornithine	29.3	0.006	–
CON-6 vs ISOL-6	Valine	20.2	0.027	–
CON-8 vs ISOL-8	1-Methylhistidine	42.7	0.156	6.18E-12
CON-8 vs ISOL-8	Adenosine triphosphate	32.4	0.041	–
CON-8 vs ISOL-8	Anserine	42.7	0.156	6.18E-12
CON-8 vs ISOL-8	Epinephrine	43.4	0.007	–
CON-8 vs ISOL-8	Glucose	-20.7	0.181	1.45E-12
CON-8 vs ISOL-8	Glycerophosphocholine	-16.8	0.020	–
CON-8 vs ISOL-8	Lactose	-13.3	0.004	–
CON-8 vs ISOL-8	Malic acid	58.1	0.055	2.35E-20
CON-8 vs ISOL-8	Methylguanidine	45.0	0.002	–
CON-8 vs ISOL-8	Saccharopine	58.1	0.055	2.35E-20
CON-8 vs ISOL-8	Unidentified	27.8	0.069	5.81E-15
CON-8 vs ISOL-8	Xylitol	-13.3	0.004	–

[1]Control-day.
[2]Corticosterone-day.
[3]Heat-day.
[4]Isolation-day.

Table S5. Percent difference, *p*-values, and metabolites found to be significantly altered in chicken breast muscle after corticosterone, heat, and isolation treatments at 2, 4, 6, and 8 days as determined by the Mann-Whitney U test (MW) or Variable Importance Analysis based on random Variable Combination analysis (VIAVC).

Treatment	Metabolite	Regulation	MW *p*-value	VIAVC *p*-value
CON-2[1] vs CORT-2[2]	Aspartate	13.5	0.311	1.26E-13
CON-2 vs CORT-2	Cystathionine	14.0	0.213	3.44E-07
CON-2 vs CORT-2	Galactonic acid	-21.0	0.201	1.34E-35
CON-2 vs CORT-2	Glucose-1-phosphate	101.5	0.069	–
CON-2 vs CORT-2	Glutamate	31.5	0.033	–
CON-2 vs CORT-2	Lactulose	-30.0	0.047	2.08E-92
CON-2 vs CORT-2	Sarcosine	23.0	0.129	6.94E-19
CON-2 vs CORT-2	Taurine	-29.8	0.057	–
CON-2 vs CORT-2	1-Methylhistidine	20.8	0.237	6.88E-23
CON-2 vs CORT-2	Carnosine	20.8	0.237	6.88E-23
CON-2 vs CORT-2	Histamine	20.8	0.237	6.88E-23
CON-2 vs CORT-2	Histidine	20.8	0.237	6.88E-23
CON-4 vs CORT-4	2-Methylbutyroylcarnitine	-28.5	0.049	–
CON-4 vs CORT-4	3-Methylhistamine	-11.7	0.078	–
CON-4 vs CORT-4	Betaine	15.4	0.248	5.56E-04
CON-4 vs CORT-4	Fructose 6-phosphate	-35.5	0.036	–
CON-4 vs CORT-4	Galactaric acid	-22.6	0.106	3.27E-03
CON-4 vs CORT-4	Glutathione	-43.8	0.240	–
CON-4 vs CORT-4	Glycerophosphorylcholine	-28.5	0.049	–
CON-4 vs CORT-4	Glycyl-glycine	18.2	0.237	1.49E-03
CON-4 vs CORT-4	Mevalonolactone	-20.0	0.040	–
CON-4 vs CORT-4	Myo-Inositol	-35.6	0.034	–
CON-4 vs CORT-4	N-Methylhydantoin	-16.9	0.041	–
CON-4 vs CORT-4	Sarcosine	24.7	0.189	4.52E-03
CON-4 vs CORT-4	Tyrosine	-39.9	0.089	3.07E-03
CON-4 vs CORT-4	Unidentified	-25.1	0.139	2.44E-03
CON-4 vs CORT-4	Unidentified	-24.5	0.096	4.47E-03
CON-4 vs CORT-4	Unidentified	-18.6	0.028	6.66E-04
CON-6 vs CORT-6	Alanine	-25.8	0.065	5.82E-15
CON-6 vs CORT-6	Betaine	-18.4	0.210	9.07E-08
CON-6 vs CORT-6	Carnitine	-7.3	0.443	4.41E-04
CON-6 vs CORT-6	Citric acid	-9.7	0.463	3.71E-04
CON-6 vs CORT-6	Glucose	5.6	0.611	4.41E-04
CON-6 vs CORT-6	Glucose-1-phosphate	7.5	0.506	1.57E-05
CON-6 vs CORT-6	Glucose-6-phosphate	37.1	0.039	4.27E-08
CON-6 vs CORT-6	Glucuronate	37.1	0.039	4.27E-08
CON-6 vs CORT-6	Glycerophosphorylcholine	-7.3	0.443	4.41E-04
CON-6 vs CORT-6	Lactate	19.8	0.344	1.49E-04
CON-6 vs CORT-6	Lactose	37.1	0.039	4.27E-08
CON-6 vs CORT-6	N,N-Dimethylglycine	-19.9	0.172	5.53E-08
CON-6 vs CORT-6	N-Methylhydantoin	-27.1	0.026	–
CON-6 vs CORT-6	Phosphonoacetate	-16.3	0.241	3.71E-04
CON-6 vs CORT-6	Unidentified	-28.1	0.047	–
CON-6 vs CORT-6	Unidentified	-35.0	0.093	2.95E-03
CON-6 vs CORT-6	Unidentified	-18.2	0.041	1.12E-03
CON-6 vs CORT-6	Unidentified	-11.8	0.066	1.42E-05
CON-8 vs CORT-8	1-Methyladenine	-56.5	0.000	3.30E-04
CON-8 vs CORT-8	Alanine	-36.9	0.011	–
CON-8 vs CORT-8	Betaine	-70.9	0.001	–

CON-8 vs CORT-8	Cystathionine	9.3	0.010	–
CON-8 vs CORT-8	Dimethylglycine	-49.2	0.003	–
CON-8 vs CORT-8	Glycine	-95.1	0.001	–
CON-8 vs CORT-8	Homocitrulline	-8.7	0.032	–
CON-8 vs CORT-8	Lactate	38.2	0.031	5.04E-33
CON-8 vs CORT-8	Sarcosine	26.1	0.016	–
CON-2 vs HEAT-2[3]	Acetamide	38.1	0.032	–
CON-2 vs HEAT-2	Cystathionine	49.7	0.014	6.30E-45
CON-2 vs HEAT-2	Glutathione	47.4	0.044	2.97E-04
CON-2 vs HEAT-2	Homocysteine	47.4	0.044	2.97E-04
CON-2 vs HEAT-2	N6-Acetyl-L-lysine	43.2	0.032	–
CON-2 vs HEAT-2	Phenylalanine	45.3	0.032	–
CON-2 vs HEAT-2	Proline	40.3	0.028	–
CON-2 vs HEAT-2	Sarcosine	40.5	0.002	4.18E-67
CON-4 vs HEAT-4	3-Hydroxyglutaric acid	99.1	0.022	–
CON-4 vs HEAT-4	4-Hydroxyproline	85.9	0.021	3.11E-09
CON-4 vs HEAT-4	Carnitine	95.4	0.034	–
CON-4 vs HEAT-4	Creatinine	17.7	0.711	9.29E-20
CON-4 vs HEAT-4	Glucose	21.0	0.259	4.53E-07
CON-4 vs HEAT-4	Lactate	-7.4	0.562	4.54E-79
CON-4 vs HEAT-4	Oxypurinol	-33.1	0.342	1.01E-05
CON-4 vs HEAT-4	Unidentified	-32.7	0.051	–
CON-4 vs HEAT-4	Unidentified	43.0	0.522	1.77E-14
CON-6 vs HEAT-6	3-Methylhistamine	-10.5	0.250	2.57E-20
CON-6 vs HEAT-6	3-Phosphoglyceric acid	-24.7	0.041	–
CON-6 vs HEAT-6	5-Thymidylic acid	-27.2	0.589	2.59E-15
CON-6 vs HEAT-6	Alanine	20.6	0.041	8.13E-34
CON-6 vs HEAT-6	Citrulline	-13.4	0.251	1.35E-13
CON-6 vs HEAT-6	Glucose	-0.7	0.941	1.50E-12
CON-6 vs HEAT-6	Hypoxanthine	-28.8	0.241	4.72E-35
CON-6 vs HEAT-6	Inosinic acid	-26.0	0.046	1.87E-10
CON-6 vs HEAT-6	Lactate	25.9	0.072	8.70E-25
CON-6 vs HEAT-6	Oxypurinol	-28.8	0.241	4.72E-35
CON-6 vs HEAT-6	Serotonin	-13.4	0.251	1.35E-13
CON-6 vs HEAT-6	Unidentified	7.5	0.690	3.62E-15
CON-8 vs HEAT-8	Galactonate	13.9	0.141	-
CON-8 vs HEAT-8	Glucuronate	13.9	0.141	-
CON-2 vs ISOL-2[4]	3-Methylhistamine	-50.8	0.010	–
CON-2 vs ISOL-2	3-Phosphoglyceric acid	-57.7	0.009	–
CON-2 vs ISOL-2	Beta-N-Acetylglucosamine	-62.6	0.005	–
CON-2 vs ISOL-2	Creatine	-39.2	0.029	–
CON-2 vs ISOL-2	Cystathionine	-52.5	0.009	–
CON-2 vs ISOL-2	Glucose	-5.9	0.685	2.31E-12
CON-2 vs ISOL-2	Glucose-1-phosphate	-88.5	0.012	–
CON-2 vs ISOL-2	Glucuronate	-39.1	0.031	–
CON-2 vs ISOL-2	Glutamate	-31.0	0.015	–
CON-2 vs ISOL-2	Lactate	20.5	0.163	3.27E-16
CON-2 vs ISOL-2	Lactulose	-30.8	0.054	6.07E-11
CON-2 vs ISOL-2	N-Formyl-L-methionine	-64.3	0.037	–
CON-2 vs ISOL-2	N-Methylhydantoin	-39.1	0.031	–
CON-2 vs ISOL-2	Pantothenic acid	-85.7	0.015	–
CON-2 vs ISOL-2	Phenylalanine	-44.7	0.013	–
CON-2 vs ISOL-2	Proline	22.4	0.140	2.05E-11
CON-2 vs ISOL-2	Thiamine pyrophosphate	-108.1	0.003	–
CON-2 vs ISOL-2	Xylulose	-95.2	0.009	–
CON-4 vs ISOL-4	Fructose 6-phosphate	32.8	0.001	7.35E-25

CON-4 vs ISOL-4	Glucose-6-phosphate	32.8	0.001	7.35E-25
CON-4 vs ISOL-4	Unidentified	16.9	0.006	–
CON-4 vs ISOL-4	Xylulose	32.8	0.001	7.35E-25
CON-6 vs ISOL-6	1,5-Anhydrosorbitol	0.7	0.909	2.88E-11
CON-6 vs ISOL-6	3-Hydroxyisovaleric acid	-27.3	0.403	1.80E-17
CON-6 vs ISOL-6	3-Methylhistidine	-39.0	0.995	2.95E-10
CON-6 vs ISOL-6	3-Methylphenylacetic acid	-13.2	0.980	2.30E-25
CON-6 vs ISOL-6	Anserine	-41.0	0.485	1.32E-11
CON-6 vs ISOL-6	Betaine	35.9	0.016	–
CON-6 vs ISOL-6	Citicoline	0.7	0.909	2.88E-11
CON-6 vs ISOL-6	Dihydrothymine	46.1	0.054	1.94E-92
CON-6 vs ISOL-6	Guanosine triphosphate	-32.1	0.842	2.13E-22
CON-6 vs ISOL-6	N,N-Dimethylglycine	18.1	0.130	2.87E-15
CON-6 vs ISOL-6	Unidentified	-24.6	0.914	1.16E-20
CON-6 vs ISOL-6	Uridine diphosphate glucose	-13.2	0.980	2.30E-25
CON-6 vs ISOL-6	Xylose	-23.4	0.297	4.23E-3
CON-8 vs ISOL-8	1-Methyladenine	-29.8	0.035	–
CON-8 vs ISOL-8	Cystathionine	20.3	0.015	4.12E-79
CON-8 vs ISOL-8	Glucuronate	33.9	0.045	7.36E-40
CON-8 vs ISOL-8	Glycerophosphorylcholine	-4.2	0.699	4.48E-18
CON-8 vs ISOL-8	Glycine	-12.2	0.484	7.79E-26
CON-8 vs ISOL-8	Inosinic acid	-6.8	0.528	3.03E-31
CON-8 vs ISOL-8	N,N-Dimethylglycine	-20.9	0.017	6.35E-72
CON-8 vs ISOL-8	Sarcosine	20.3	0.015	4.12E-79
CON-8 vs ISOL-8	Sedoheptulose	-4.2	0.699	4.48E-18
CON-8 vs ISOL-8	Taurine	-40.1	0.051	–
CON-8 vs ISOL-8	Unidentified	36.0	0.029	–

[1]Control-day.
[2]Corticosterone-day.
[3]Heat-day.
[4]Isolation-day.

Table S6. Percent difference, *p*-values, and metabolites found to be significantly altered in chicken hippocampus after corticosterone, heat, and isolation treatments at 2, 4, 6, and 8 days as determined by the Mann-Whitney U test (MW) or Variable Importance Analysis based on random Variable Combination analysis (VIAVC).

Treatment	Metabolite	Regulation	MW *p*-value	VIAVC *p*-value
CON-2[1] vs CORT-2[2]	Ascorbate	6.1	0.024	–
CON-2 vs CORT-2	Citric acid	-20.2	0.025	3.46E-68
CON-2 vs CORT-2	Myo-Inositol	4.7	0.090	–
CON-2 vs CORT-2	Acetylcholine	6.1	0.024	–
CON-2 vs CORT-2	Taurine	4.7	0.090	–
CON-4 vs CORT-4	1-Methylhistidine	-10.5	0.002	–
CON-4 vs CORT-4	Acetylcarnitine	-21.5	0.022	–
CON-4 vs CORT-4	Adenosine monophosphate	-8.7	0.027	–
CON-4 vs CORT-4	Adenosine phosphosulfate	-16.5	0.024	–
CON-4 vs CORT-4	Carnitine	-28.0	0.017	–
CON-4 vs CORT-4	Citric acid	-14.3	0.033	–
CON-4 vs CORT-4	Creatine	18.5	0.010	–
CON-4 vs CORT-4	Dimethylglycine	33.4	0.041	–
CON-4 vs CORT-4	Galactaric acid	-11.6	0.033	–
CON-4 vs CORT-4	Glutamate	-12.6	0.023	2.10E-42
CON-4 vs CORT-4	Glutamine	-21.5	0.020	1.40E-34
CON-4 vs CORT-4	Hydroxyacetone	-11.4	0.027	–
CON-4 vs CORT-4	Lactose	-7.8	0.029	–
CON-4 vs CORT-4	Levulinate	-9.0	0.041	–
CON-4 vs CORT-4	Myo-Inositol	-6.2	0.037	–
CON-4 vs CORT-4	N-Acetylaspartate	-13.5	0.015	–
CON-4 vs CORT-4	N-Acetylgalactosamine 4-sulphate	-16.5	0.024	–
CON-4 vs CORT-4	N-Acetylornithine	-26.7	0.066	–
CON-4 vs CORT-4	Phenylephrine	-23.4	0.024	–
CON-4 vs CORT-4	Phosphocholine	-26.7	0.066	–
CON-4 vs CORT-4	Pyruvic acid	-26.1	0.030	–
CON-4 vs CORT-4	Uridine monophosphate	-17.8	0.032	–
CON-4 vs CORT-4	Xylulose	-12.9	0.026	–
CON-6 vs CORT-6	Adenosine monophosphate	15.1	0.015	1.10E-10
CON-6 vs CORT-6	Aspartic acid	14.9	0.044	–
CON-6 vs CORT-6	Erythritol	9.3	0.035	–
CON-6 vs CORT-6	Ethanol	8.0	0.036	–
CON-6 vs CORT-6	Ethylene glycol	8.0	0.036	–
CON-6 vs CORT-6	FAPy-adenine	-57.0	0.065	–
CON-6 vs CORT-6	Gluconic acid	8.0	0.036	–
CON-6 vs CORT-6	Gluconolactone	8.0	0.036	–
CON-6 vs CORT-6	Glycerophosphocholine	9.3	0.030	–
CON-6 vs CORT-6	Lactate	15.6	0.026	–
CON-6 vs CORT-6	Lactulose	9.3	0.035	–
CON-6 vs CORT-6	Mannitol	9.3	0.035	–
CON-6 vs CORT-6	Threonate	10.5	0.033	–
CON-6 vs CORT-6	Tyrosine	-39.5	0.106	8.39E-11
CON-8 vs CORT-8	Creatine	29.5	0.013	–
CON-8 vs CORT-8	Cysteine	-11.6	0.049	–
CON-8 vs CORT-8	Diethanolamine	-19.5	0.041	–
CON-8 vs CORT-8	Glutamate	-3.8	0.310	1.98E-14
CON-8 vs CORT-8	Glycerophosphocholine	-19.5	0.041	–
CON-8 vs CORT-8	Guanidoacetic acid	-15.3	0.091	–
CON-8 vs CORT-8	N-Acetylaspartate	-1.1	0.818	6.35E-86
CON-8 vs CORT-8	Tyrosine	-64.5	0.040	1.60E-28

CON-8 vs CORT-8	Uridine monophosphate	-24.4	0.049	–
CON-2 vs HEAT-2[3]	3-Methyladenine	-7.3	0.136	4.94E-40
CON-2 vs HEAT-2	4-Aminobutyrate	-14.3	0.046	–
CON-2 vs HEAT-2	6-Phosphogluconic acid	-7.3	0.136	4.94E-40
CON-2 vs HEAT-2	Adenosine monophosphate	19.6	0.041	–
CON-2 vs HEAT-2	Anserine	9.3	0.227	6.23E-22
CON-2 vs HEAT-2	Creatine	-7.3	0.136	4.94E-40
CON-2 vs HEAT-2	Creatinine	-28.5	0.055	–
CON-2 vs HEAT-2	Dimethylamine	11.0	0.033	2.32E-35
CON-2 vs HEAT-2	Erythritol	-2.8	0.701	7.92E-43
CON-2 vs HEAT-2	Ethanol	-1.9	0.771	1.58E-42
CON-2 vs HEAT-2	Ethylene glycol	-1.9	0.771	1.58E-42
CON-2 vs HEAT-2	Gluconic acid	-1.9	0.771	1.58E-42
CON-2 vs HEAT-2	Gluconolactone	-1.9	0.771	1.58E-42
CON-2 vs HEAT-2	Glutaric acid	-14.3	0.046	–
CON-2 vs HEAT-2	Glycerophosphocholine	-2.8	0.701	7.92E-43
CON-2 vs HEAT-2	Lactate	-7.4	0.034	–
CON-2 vs HEAT-2	Lactulose	-2.8	0.701	7.92E-43
CON-2 vs HEAT-2	L-Phenylalanine	-7.3	0.136	4.94E-40
CON-2 vs HEAT-2	Mannitol	-2.8	0.701	7.92E-43
CON-2 vs HEAT-2	Myo-Inositol	-28.5	0.055	–
CON-2 vs HEAT-2	N-Acetylaspartate	11.0	0.033	2.32E-35
CON-2 vs HEAT-2	N-Acetylgalactosamine 4-sulphate	-28.5	0.055	–
CON-2 vs HEAT-2	N-Acetylglutamate	-14.3	0.046	–
CON-2 vs HEAT-2	Niacinamide	19.6	0.041	–
CON-2 vs HEAT-2	N-Methylhydantoin	-7.4	0.034	–
CON-2 vs HEAT-2	Phosphorylcholine	-21.9	0.009	3.07E-46
CON-2 vs HEAT-2	Quinic acid	-14.3	0.046	–
CON-2 vs HEAT-2	Threonate	-5.5	0.384	2.47E-40
CON-2 vs HEAT-2	Tyrosine	-21.9	0.009	3.07E-46
CON-4 vs HEAT-4	Adenosine monophosphate	-14.2	0.128	1.67E-31
CON-4 vs HEAT-4	Adenosine triphosphate	-5.2	0.632	3.43E-32
CON-4 vs HEAT-4	Ascorbate	-15.3	0.240	1.67E-31
CON-4 vs HEAT-4	Guanosine	-8.2	0.526	1.56E-48
CON-4 vs HEAT-4	Niacinamide	19.4	0.702	2.21E-18
CON-4 vs HEAT-4	Phenylalanine	33.7	0.305	6.47E-27
CON-4 vs HEAT-4	Xylulose	-9.9	0.371	2.24E-58
CON-6 vs HEAT-6	3-Methoxytyramine	14.8	0.015	–
CON-6 vs HEAT-6	5-Methoxytryptamine	13.1	0.363	2.69E-20
CON-6 vs HEAT-6	Aspartic acid	15.5	0.032	–
CON-6 vs HEAT-6	Cystathionine	15.0	0.026	7.24E-21
CON-6 vs HEAT-6	Dimethylamine	15.0	0.026	7.24E-21
CON-6 vs HEAT-6	Erythritol	14.8	0.021	–
CON-6 vs HEAT-6	Glycerophosphocholine	13.5	0.096	6.17E-09
CON-6 vs HEAT-6	Guanidinosuccinic acid	14.4	0.065	3.73E-13
CON-6 vs HEAT-6	Lactulose	14.8	0.021	–
CON-6 vs HEAT-6	Mannitol	14.8	0.021	–
CON-6 vs HEAT-6	Methanol	15.2	0.303	2.07E-10
CON-6 vs HEAT-6	Phenylephrine	12.2	0.260	5.92E-31
CON-6 vs HEAT-6	Succinic acid semialdehyde	15.0	0.026	7.24E-21
CON-6 vs HEAT-6	Sucrose	19.4	0.035	–
CON-6 vs HEAT-6	Threonate	14.8	0.021	–
CON-6 vs HEAT-6	Unidentified	30.5	0.324	1.30E-13
CON-8 vs HEAT-8	Alanine	-2.2	0.768	5.88E-12
CON-8 vs HEAT-8	Anserine	-20.2	0.030	–
CON-8 vs HEAT-8	Cysteine	-14.7	0.014	–

CON-8 vs HEAT-8	Glutamate	5.7	0.557	3.21E-15
CON-8 vs HEAT-8	Glutamine	-2.2	0.768	5.88E-12
CON-8 vs HEAT-8	Glycine	-12.3	0.018	–
CON-8 vs HEAT-8	Guanidoacetic acid	-17.2	0.033	–
CON-8 vs HEAT-8	Myo-Inositol	-3.5	0.737	2.51E-33
CON-8 vs HEAT-8	S-Adenosylhomocysteine	-21.5	0.023	–
CON-8 vs HEAT-8	Valine	-26.9	0.163	6.54E-12
CON-8 vs HEAT-8	Xylulose	-15.6	0.011	–
CON-2 vs ISOL-2[4]	2,3-Butanediol	-51.1	0.040	–
CON-2 vs ISOL-2	3-Phenyllactate	-11.0	0.020	–
CON-2 vs ISOL-2	4-Aminobutyrate	-13.0	0.029	–
CON-2 vs ISOL-2	4-Methylcatechol	-13.0	0.022	–
CON-2 vs ISOL-2	Aspartate	-12.2	0.015	–
CON-2 vs ISOL-2	Canthaxanthin	-13.0	0.029	–
CON-2 vs ISOL-2	Citramalic acid	-16.7	0.018	–
CON-2 vs ISOL-2	Citric acid	-26.2	0.011	–
CON-2 vs ISOL-2	Creatinine	-31.3	0.015	–
CON-2 vs ISOL-2	Cystathionine	-10.0	0.076	5.13E-03
CON-2 vs ISOL-2	Diethanolamine	-19.5	0.054	6.18E-03
CON-2 vs ISOL-2	Epinephrine	-16.7	0.018	–
CON-2 vs ISOL-2	Ethanol	-51.1	0.040	–
CON-2 vs ISOL-2	Glycerophosphocholine	-10.5	0.288	6.48E-03
CON-2 vs ISOL-2	Isopropanol	-51.1	0.040	–
CON-2 vs ISOL-2	Lactulose	-28.4	0.036	–
CON-2 vs ISOL-2	Malate	-12.2	0.015	–
CON-2 vs ISOL-2	Myo-Inositol	-27.7	0.038	2.59E-03
CON-2 vs ISOL-2	N-Acetylgalactosamine 4-sulphate	-31.3	0.015	–
CON-2 vs ISOL-2	N-Acetylglutamate	-13.0	0.026	–
CON-2 vs ISOL-2	Phenylalanine	-8.9	0.080	5.13E-03
CON-2 vs ISOL-2	Phosphorylcholine	-25.9	0.050	2.59E-03
CON-2 vs ISOL-2	S-Adenosylhomocysteine	-12.2	0.015	–
CON-2 vs ISOL-2	Selenomethionine	-13.0	0.022	–
CON-2 vs ISOL-2	Succinic acid semialdehyde	-13.0	0.012	–
CON-2 vs ISOL-2	Thymine	-13.0	0.029	–
CON-2 vs ISOL-2	Unidentified	-33.4	0.011	–
CON-4 vs ISOL-4	4-Methylcatechol	11.5	0.049	–
CON-4 vs ISOL-4	Adenosine monophosphate	-11.6	0.008	4.98E-57
CON-4 vs ISOL-4	Aspartic acid	-6.3	0.060	1.20E-54
CON-4 vs ISOL-4	Creatinine	17.9	0.012	–
CON-4 vs ISOL-4	Glutamine	6.9	0.057	4.94E-41
CON-4 vs ISOL-4	Glutathione	97.7	0.052	–
CON-4 vs ISOL-4	Lactulose	9.8	0.065	–
CON-4 vs ISOL-4	Myo-Inositol	13.8	0.039	–
CON-4 vs ISOL-4	N-Acetylgalactosamine 4-sulphate	17.9	0.012	–
CON-4 vs ISOL-4	N-Acetylglutamate	11.5	0.049	–
CON-4 vs ISOL-4	Oxypurinol	17.6	0.178	3.65E-62
CON-4 vs ISOL-4	Phosphorylcholine	9.8	0.065	–
CON-4 vs ISOL-4	Selenomethionine	11.5	0.049	–
CON-4 vs ISOL-4	UDP-glucose	15.5	0.388	9.57E-46
CON-6 vs ISOL-6	3-Methylhistamine	15.7	0.099	–
CON-6 vs ISOL-6	4-Aminobutyrate	19.6	0.041	–
CON-6 vs ISOL-6	Glycerophosphocholine	14.2	0.026	–
CON-6 vs ISOL-6	Isoleucine	-58.3	0.030	–
CON-6 vs ISOL-6	Lactate	11.4	0.240	1.33E-27
CON-6 vs ISOL-6	L-Aspartic acid	13.0	0.045	–
CON-6 vs ISOL-6	Myo-Inositol	11.3	0.180	1.64E-27

CON-6 vs ISOL-6	Unidentified	80.5	0.005	2.20E-89
CON-6 vs ISOL-6	Unidentified	30.7	0.064	–
CON-8 vs ISOL-8	1-Methylhistidine	-23.3	0.009	–
CON-8 vs ISOL-8	1-Methylnicotinamide	-25.0	0.012	–
CON-8 vs ISOL-8	3-Methoxybenzenepropanoic acid	-25.4	0.021	–
CON-8 vs ISOL-8	3-Methoxytyrosine	-28.3	0.006	–
CON-8 vs ISOL-8	3-Methylhistamine	-25.6	0.007	–
CON-8 vs ISOL-8	4-Hydroxyproline	-46.3	0.022	–
CON-8 vs ISOL-8	7-Methylguanosine	-50.1	0.003	–
CON-8 vs ISOL-8	Acetylcarnitine	-32.2	0.025	–
CON-8 vs ISOL-8	Adenosine phosphosulfate	-24.4	0.026	–
CON-8 vs ISOL-8	Adenosine triphosphate	-38.7	0.013	–
CON-8 vs ISOL-8	Anserine	-22.0	0.011	–
CON-8 vs ISOL-8	FAPy-adenine	-91.8	0.027	–
CON-8 vs ISOL-8	Galactaric acid	-29.9	0.010	–
CON-8 vs ISOL-8	Glutathione	43.0	0.033	1.1E-07
CON-8 vs ISOL-8	Glycerophosphocholine	-30.5	0.022	8.8E-05
CON-8 vs ISOL-8	Guanosine	-51.73	0.007	–
CON-8 vs ISOL-8	Homoveratric acid	38.4	0.041	–
CON-8 vs ISOL-8	Inosine	-35.0	0.024	–
CON-8 vs ISOL-8	Malate	-37.4	0.020	–
CON-8 vs ISOL-8	Methanol	-36.5	0.021	–
CON-8 vs ISOL-8	N-Acetylaspartate	-25.1	0.016	–
CON-8 vs ISOL-8	N-Acetylgalactosamine	-49.3	0.012	–
CON-8 vs ISOL-8	N-Acetylgalactosamine 4-sulphate	-24.4	0.026	–
CON-8 vs ISOL-8	N-Acetylmannosamine	71.0	0.015	–
CON-8 vs ISOL-8	N-Acetylornithine	-47.6	0.005	–
CON-8 vs ISOL-8	Niacinamide	-68.2	0.025	–
CON-8 vs ISOL-8	Phenylephrine	-39.9	0.003	6.7E-06
CON-8 vs ISOL-8	Phosphocholine	-49.8	0.008	–
CON-8 vs ISOL-8	S-Adenosylhomocysteine	-24.4	0.019	–
CON-8 vs ISOL-8	Sucrose	-29.4	0.012	–
CON-8 vs ISOL-8	Threonate	-44.1	0.002	–
CON-8 vs ISOL-8	Threonine	-27.7	0.035	–
CON-8 vs ISOL-8	Trigonelline	-61.9	0.007	–
CON-8 vs ISOL-8	Tyrosine	30.8	0.019	–
CON-8 vs ISOL-8	Unidentified	-43.4	0.005	–
CON-8 vs ISOL-8	Unidentified	-52.3	0.008	–
CON-8 vs ISOL-8	Unidentified	-54.1	0.014	–
CON-8 vs ISOL-8	Unidentified	-48.8	0.002	–
CON-8 vs ISOL-8	Unidentified	41.9	0.048	–
CON-8 vs ISOL-8	Unidentified	-59.7	0.041	–
CON-8 vs ISOL-8	Unidentified	19.1	0.034	–
CON-8 vs ISOL-8	Unidentified	-81.9	0.022	–
CON-8 vs ISOL-8	Uridine 5'-diphosphate	-87.4	0.033	–
CON-8 vs ISOL-8	Uridine monophosphate	-27.2	0.018	–
CON-8 vs ISOL-8	Xylulose	-44.3	0.006	–

[1]Control-day.
[2]Corticosterone-day.
[3]Heat-day.
[4]Isolation-day.
